# The aadE*-*sat4*-*aphA-3* Gene Cluster of *Mycoplasma bovirhinis* HAZ141_2 Undergoes Genomic Rearrangements Influencing the Primary Promoter Sequence

**DOI:** 10.3390/antibiotics10111335

**Published:** 2021-11-01

**Authors:** Inna Lysnyansky, Ilya Borovok

**Affiliations:** 1 Mycoplasma Unit, Division of Avian Diseases, Kimron Veterinary Institute, Beit Dagan 50250, Israel; 2 The Shmunis School of Biomedicine and Cancer Research, George S. Wise Faculty of Life Sciences, Tel-Aviv University, Tel Aviv 69978, Israel

**Keywords:** *Mycoplasma*, kanamycin, neomycin, nourseothricin, resistance, prophage, *aadE*-*sat4*-*aphA-3*, inversions, deletions, promoters

## Abstract

The 54 kb GC-rich prophage region of *Mycoplasma bovirhinis* HAZ141_2 contains three structural ‘compartments’, one of which is a highly transmittable cluster of three genes, *aadE*-like (aadE*), *sat4*, and *aphA-3*. In this study, we characterized recombination events and their consequences occurred within the aadE*-*sat4*-*aphA-3* containing region. Analysis of this region revealed direct repeats (DRs) of 155 and invert repeats (IRs) of 197 base pairs (bps) each, flanking and overlapping with the primary promoter P* located upstream of the aadE*. Two recombination events, including inversions via both 197 and 155-bp IRs (the latter become inverted after the initial 197-bp IRs associated inversion) and the excision of the aadE*-*sat4*-*aphA-3* cluster, were confirmed. Inversion via 155-IRs results in changes within the P* promoter region. Using *Escherichia coli* JM109 carrying plasmids containing derivatives of the aadE*-*sat4*-*aphA-3* cluster, we validated the expression of those genes from different promoters. Our results showed no difference in the minimal inhibitory concentrations (MICs) to kanamycin and neomycin and only 2-fold decrease in MIC (from 512 to 256 μg/mL) to nourseothricin between the wild type and a P* derivative promoter. However, the MICs to kanamycin and neomycin were at least 4-fold lower in the construct where *aphA-3* expressed under its P2 promoter (128 µg/mL) in comparison to the construct where *aphA-3* expressed under P1″ promoter located within the *sat4* gene (512–1024 µg/mL). PCR confirmed the excision of the aadE*-*sat4*-*aphA-3* cluster via 197- and 155-bp DRs, but no selection of antibiotic-sensitive *M. bovirhinis* were obtained after 100 passages in kanamycin-free medium.

## 1. Introduction

Recently, a 53,923 bp prophage-like genomic region was identified and characterized in the genome of *Mycoplasma bovirhinis* HAZ141_2 isolated in Japan from bovine nasal discharge [1,2,3]. *M. bovirhinis* is a bovine *Mycoplasma* species found worldwide; it is frequently isolated from cattle with respiratory disease as well as from healthy asymptomatic carriers [4]. Several features of *M. bovirhinis* HAZ141_2 prophage, like high GC content (40.7%) in contrast to the core genome (28.24%) and almost solely using TGG, but not TGA codon to encode amino acid (aa) tryptophan (Trp) highlighted horizontal gene transfer (HGT) and recent exogenous acquisition of this prophage by *M. bovirhinis* [3]. The *M. bovirhinis* HAZ141_2 prophage-like region has a modular structural organization with *aadE*-like (aadE*), *sat4*, and *aphA-3* genes located at its 5′ end [3]. The *aadE-sat4-aphA-3* gene cluster encodes to 6′ adenyltransferase [AAD(6′)], N-acetyltransferase and 3′ phosphotransferase [APH(3′)-III] conferring resistance to streptomycin (Sm), streptothricin/nourseothricin (NTC), and to kanamycin (Kn)/neomycin (Nm), respectively [5]. The *aadE-sat4-aphA-3* genes have been detected within the chromosome of *Campylobacter coli* [6] as well as on transposons, plasmids, and phages of both Gram-positive and Gram-negative bacteria [7,8,9,10] underlining its high transmittable nature. Remarkably, while *M. bovirhinis* HAZ141_2 possessed high-level resistance to Kn/Nm (≥512 µg/mL), its minimal inhibitory concentration values (MICs) to NTC (16–32 µg/mL) and streptomycin (64 µg/mL) were compatible with MICs for the same antibiotics obtained for prophageless *M. bovirhinis* type strain PG43 and Israeli filed isolate 316,981 [3]. However, when the aadE*-*sat4*-*aphA-3* genes were cloned into a low-copy number vector and transferred into antibiotic-sensitive *Escherichia coli* JM109 cells, high MICs to Kn/Nm and NTC (≥256 µg/mL), but still not to Sm, have been evident. The results of RT-PCR showed co-transcription of *M. bovirhinis* HAZ141_2 aadE*, *sat4*, and *aphA-3* genes from the common primary promoter (P*), identified 41 bp upstream of the ATG start codon of the aadE* gene. In addition, we predicted in silico two other putative promoters supporting *aphA-3* expression—P1″, located within encoding sequence of *sat4*, and P2, located 34 bp upstream of the ATG start codon of *aphA-3* [3]. Predicted P1″ and P2 promoters resemble, respectively, P1/P1′ and P2 promoters of *aphA-3*, which have been previously characterized in other bacteria [11,12,13].

The present study reports that under standard laboratory growth conditions, the *M. bovirhinis* HAZ141_2 genomic region containing aadE*-sat4-aphA-3 undergoes spontaneous recombination events, which include inversions as well as deletions of these antibiotic resistance genes via both 197- and 155-bp repeated DNA sequences that flank them. Inversion via 155-bp results in changes within the native P* promoter region. To further investigate the potential of such changes, the expression of the aadE*-sat4-aphA3-gene cluster from different promoters (P* corresponds to the genetic configuration found during the genome sequencing of the *M. bovirhinis* strain HAZ141_2, derivate P* (P*^Der^), obtained after inversion through 155-bp IRs as well as P1″, and P2) were tested in *E. coli* JM109 cells by measuring MICs to Kn, Nm, and NTC.

## 2. Results

### 2.1. Different Types of Repetitive Sequences Flank and Overlap with the Primary P* Promoter of the aadE*-sat4-aphA-3 Gene Cluster

**Figure 1:**
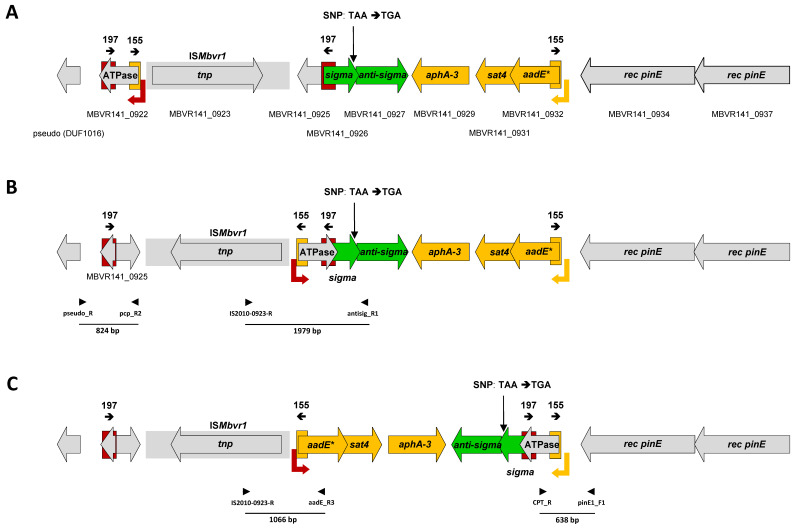
Schematic representation of the inversions occurring within and near the *M. bovirhinis* HAZ141-2 aadE*-*sat4*-*aphA-3* gene cluster. Genomic organization of the region containing aadE*-*sat4*-*aphA-3*, found during the genome sequencing of the *M. bovirhinis* HAZ141-2 (**A**), the same region after inversion via 197 bp long IRs (**B**) and after inversion via 155 bp long IRs (**C**). Arrows represent open reading frames (ORFs) with the arrowhead indicating the direction of transcription. The ORFs are color coded based on the putative function of the predicted encoded proteins as follows: yellow—antibiotic resistance genes; green—transcriptional regulation genes; grey—rest of the genes. The grey shaded region in the back of IS element includes IS*Mbvr1-*encoding ORF as well as IS-related sequences. The genes are tagged according to GenBank AP018135.1 [1]. The 197 and 155 bp long repeats are marked by red and yellow rectangles, respectively, and their length and direction are shown above. The P* and P*^Der^ promoter-associated regions are represented by yellow and red downwards arrows, respectively, with corners pointing in the direction of transcription.

Sequence analysis of the prophage region containing aadE*-*sat4*-*aphA-3* gene cluster revealed presence of two types of repeated sequences: invert and direct repeats (IRs and DRs, respectively) (Figure 1). The IRs are 197 bp long and differ by four mismatches (Figure S1). The IRL located at 3′ of the MBOV141_0922 (nc 870715-870911; nucleotide coordinates are shown hereinafter in the text according to GenBank AP018135.1; [1]) encoding a putative AAA-ATPase, while the IRR located at the 5′ of the MBOV141_0926 (nc 873765-873961) encodes a putative DNA-directed RNA polymerase alternative sigma factor (Figure 1). The DRs are 155 bp long, have two mismatches (Figure S1), and are located at the 5′ of the MBOV141_0922 (nc 871101-871255; DRL) as well as at 5′ of the aadE* (MBOV141_0932; 876931-877085; DRR) genes. Notably, the 155-bp-DRR is overlapped with the primary promoter P* located upstream of the aadE* gene (Figure 1).

The primers used for PCR amplifications to confirm the new received junctions after inversions are shown by arrowheads and black bars, respectively, and the sizes of the PCR products are shown. Location of a single nucleotide polymorphism (SNP) that changed TAA (stop codon) to TGA (Trp) and caused an extension of MBVR141_0926 C-terminus by additional 60 aa is indicated by black vertical arrow. Schematic representation of this genomic region has been prepared using IS*Mbvr1* (2010 bp) as a scale bar.

### 2.2. Recombination between IRs Results in Genomic Inversions of the aadE*-sat4-aphA-3 Gene-Containing Regions

We hypothesized that the IR sequences identified within and near the aadE*-*sat4*-*aphA-3* cluster serve as the targets for recombination that result in inversions leading to rearrangements of this region. The PCR amplifications and Sanger sequencing of the resulting products were undertaken to verify this hypothesis. Initially, we tested whether inversion of a 3050 bp genomic fragment may occur through 197-bp long IRs (Figure 1A,B). Sequence analysis of 824-bp and 1979-bp predicted PCR amplicons confirmed the assumed junctions (Figure 1B). The inversion via 197-bp IRs leads to extension of the MBVR141_0926 ORF from 162 to 328 amino acids (aa) (Figure S2). This elongation was a result of the two events: (i) fusion of MBVR141_0926 to MBVR141_0922 that extended N terminus of the former by 106 aa, (ii) the presence of a single nucleotide polymorphism (SNP) that changed TAA (stop codon, nc 874248-874250) to TGA (encodes Trp in *Mycoplasma* species) and caused extension of MBVR141_0926 C-terminus by additional 60 aa. To clarify which sequence, regarding the SNP, was found in the genomic DNA of *M. bovirhinis* HAZ141_2 isolate present in our laboratory, several independent and different PCR reactions were performed, and their products were subjected to Sanger sequencing (data not shown). The sequence data confirmed a presence of TGA codon in place of TAA within MBVR141_0926 of the strain found in our laboratory; such SNP ‘eliminated’ the annotated stop codon and resulted in a 222 aa protein in length with a new stop codon (874428-874430) (Figure 1). Regardless of the SNP (converting TAA to TGA), inversion via 197-bp IRs caused extension of the N-terminal part of the MBVR141_0926 by 106 aa as mentioned above. The short versions of the protein (either 162 or 222 aa) possess only RNA polymerase sigma-70 factor domain (TIGR02937), while the extended version (328 aa) also contains an AAA-ATPases domain (pfam13238) [3]. In addition, in the short versions the predicted start codon for translation of MBVR141_0926 is TTG (formally encodes Leu but it is also known as coding for fMet), while in the extended version it is ATA (formally encodes Ile but also known as an alternative start codon; (Figure S2)) [14]. Notably, while a 162 aa encoding version of the MBVR141_0926 overlaps the downstream located MBVR141_0927, encoding putative anti-sigma factor, by 19 bp, both 222 and 328 aa encoding versions overlap it by 199 bp (data not shown). The functional relevance of such overlapping as well as MBVR141_0926 extension were not tested in this study and meanwhile remains unclear.

**Figure 2:**
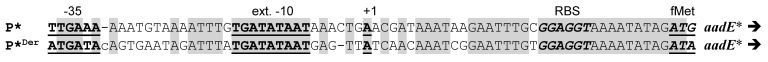
Comparison of two replaceable promoter regions identified upstream of the aadE* gene of *Mycoplasma bovirhinis* HAZ141_2. Nucleotide sequence alignment of the promoter region before and after inversion via 155 bp IRs (P* and P*^Der^, respectively) was performed using the public server running the ClustalW program (https://npsa-prabi.ibcp.fr/cgi-bin/npsa_automat.pl?page=/NPSA/npsa_clustalwan.html (accessed on 29 September 2021); [16]). Promoter elements, as previously identified for P* [3], are shown as -35 and ext. -10 (extended -10) above the underlined nucleotides in bold. The 5′RACE experimentally validated transcription start site of P* and that predicted for P*^Der^ are shown in bold and underlined as well as marked with +1. Predicted ribosome-binding site (RBS) is shown in bold italic. Predicted first (‘start’) codons of aadE* before (ATG) and after inversion (ATA) that both code for formyl-methionine (fMet) are shown in bold italic and underlined. The arrow after the gene name (aadE*) indicates the direction of transcription. Identical nucleotides are gray highlighted.

As a result of the initial 197-bp IRs associated inversion, 155-bp long DRs become be inverted (Figure 1B). Therefore, we tested if an inversion of a 3511 bp DNA fragment, located between these newly established 155-bp IRs may occur. Sequence analysis of 1066 and 638 bp predicted PCR amplicons confirmed the newly obtained junctions (Figure 1C). An inversion via 155 bp established IRs performed several sequence changes within the former P* promoter region (P*^Der^) including the replacement of ATG start codon to ATA, which can be used as an alternate start codon [14,15] (Figure 2). The BLASTN analysis using 73 bp P*^Der^ promoter sequence (Figure S3) as a query revealed totally identical sequences found upstream of aadE* genes located on the plasmids of *Campylobacter coli* as well as *Campylobacter jejuni*, while the search performed with the P*^WT^ sequence revealed its uniqueness at the NCBI database (September 2021).

### 2.3. In Vitro Validation of the aadE*-sat4-aphA-3 Expression from Different Promoters

Further, we tested if changes within the P* promoter region (Figure 2 and Figure S3), that occurred after inversion via 155-bp IRs, influenced the expression of the aadE*-*sat4*-*aphA-3* genes. Using *E. coli* JM109 as a heterologous host, we constructed several plasmids containing structural derivatives of the aadE*-*sat4*-*aphA-3* genes (Figures S4 and S5, Tables S1 and S2) as described in Materials and Methods. First, to avoid the effect of the multiple promoters/transcriptional initiation sites of pACYC184 [17], a vector derivate lacking *tet* gene as well as P2 and P4 plasmid promoters (to distinguish between P2 promoter of pACYC184 plasmid and P2 promoter of the of *aphA-3* gene, we will use P2^vec^ designation for the former) was constructed (pACYC_∆tet∆P2^vec^∆P4^vec^, Figure S4, Table S1 and Materials and Methods). Second, since *Hind*III and *Sph*I restriction sites, used for the cloning, located downstream of the *cat* gene, we verified if there is any influence of P5 *cat*-related promoter [17] on the expression of the experimental constructs. For this, the P2-promoterless *aphA-3* gene (Figure S5) was cloned into pACYC_∆tet∆P2^vec^∆P4^vec^ derivate in the same orientation as the *cat* gene (pACYC_∆P2_aphA3-17) or in an orientation opposite of the *cat* gene (pACYC_∆P2_aphA3-19). The expression of the *aphA-3* gene in these clones, assessed by their susceptibility to Kn/Nm, demonstrated a clear effect of the P5 promoter on *aphA-3* expression (Table 1).

Indeed, in comparison to pACYC184 plasmid, used as a control, the 256-512-fold increase in the MICs to Kn/Nm was obtained in the case of pACYC_∆P2_aphA3-17 (1 vs. 256–512 μg/mL), while only a slight increase (1 vs. 4 μg/mL) was found for pACYC_∆P2_aphA3-19 (Table 1). Based on this experience, all subsequent constructs were performed by cloning genes of our interest in the orientation opposite of *cat* gene (Figures S4 and S5, Table S2). Finally, expression of the aadE*-*sat4*-*aphA-3* gene cluster derivatives from the different promoters, was evaluated through the determination of their MIC values to Kn/Nm and NTC (Table 1). With exception of pACYC_P2_aphA3, no differences in MIC values to Kn/Nm were found among the constructs. The pACYC_P2_aphA3 construct, in which the *aphA-3* gene was cloned with its predicted P2 promoter, demonstrated at least 4-fold decrease in MICs to Kn/Nm in comparison to the constructs, where *aphA-3* is located under three promoters (P*, P1″, and P2) or under P1″ promoter alone (Table 1). Only a 2-fold decrease in MIC to NTC (from 512 to 256 μg/mL) was found between pACYC_P* and pACYC_P*^Der^, obtained after inversion via 155-bp IRs (Table 1 and Figure 1C).

### 2.4. Competency for Excision of the aadE*-sat4-aphA-3 Gene Cluster

**Figure 3:**
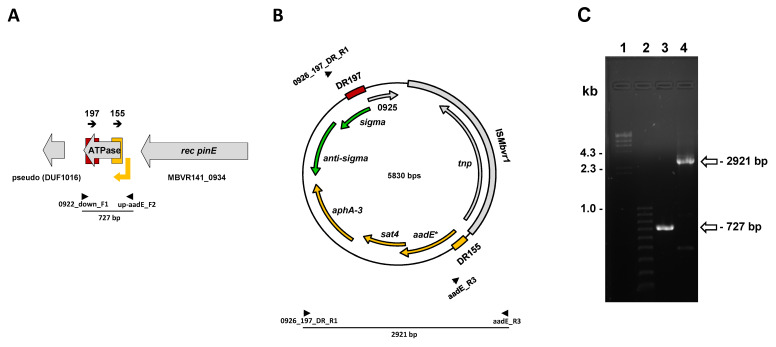
The excision (deletion) and circularization of the *M. bovirhinis* HAZ141_2 aadE*-*sat4*-*aphA-3* gene cluster. Schematic representation of the excision (**A**) and circularization (**B**) of the gene cluster that occurred either by recombination between 155-bp DRs (Figure 1A) or by recombination between 197-bp DRs, established after inversion via 155-bp IRs (Figure 1C). In both cases, the resulting sequences of the deleted as well as circular products are almost identical. Arrows represent open reading frames (ORFs) with the arrowhead indicating the direction of transcription. The ORFs are color coded based on the putative function of the predicted encoded proteins as follows: yellow—antibiotic resistance genes; green—transcriptional regulation genes; grey—rest of the genes. A *tnp* (MBVR141_0923) is encoding transposase IS*Mbvr1*; an aadE* (MBVR141_0932) is identical homologue of aadE* having an AUA codon as the 1st one; *sat4* (MBVR141_0931), *aphA-3* (MBVR141_0929), *anti-sigma* (MBVR141_0927), *sigma* (MBVR141_0926), and 0925 (MBVR141_0925) are described in the text. Both 197 and 155 bp long repeats are marked as direct repeats (DR197 and DR155, respectively) according to their genomic direction (orientation) in eve of an excision event. The P* promoter-associated region is represented by yellow colored downwards arrow with the corner pointing in the direction of transcription (**A**). The primers used to confirm a deletion (0922_down_F1 and up_aadE_F2) or a circularization (0926_197_DR_R1 and aadE_R3) of the aadE*-*sat4*-*aphA-3* gene cluster are shown by arrowheads and black bars; the size of the PCR products is shown below the bars. (**C**) Agarose gel electrophoresis of PCR products showing excision (lane 3) and circularization (lane 4) of the aadE*-*sat4*-*aphA-3* gene cluster either through 155-bp DRs (WT configuration; Figure 1A) or through 197-bp DRs, established after inversion via 155-bp IRs (Figure 1C) or through both of them. The size of the PCR products, received with 0922_down_F1 and up_aadE_F2 primers (lane 3) and with 0926_197_DR_R1 and aadE_R3 (lane 4), is shown by arrows. The λ-*Hind*III (Thermo Scientific™, Waltham, MA, USA) and 100-bp ladder (BioRad, Hercules, CA, USA) are shown in lanes 1 and 2, respectively.

At least two possible scenarios of excision (deletion) and circularization of the gene cluster can be assumed (Figure 3). First is by recombination between 155-bp DRs (Figure 1A), and the second one by recombination between 197-bp DRs, established after inversion via 155-bp IRs (Figure 1C). Since in both cases described above, the resulted sequences of the deleted as well as circular products were almost identical (Figure 3A,B), it was impossible to design specific PCR systems to confirm separately each scenario. Therefore, the PCR amplification products do support the excision and circularization of the aadE*-*sat4*-*aphA-3* gene cluster (Figure 3C) but cannot point out if they are a result of inversion via recombination between 155-bp DRs or between 197-bp DRs or both.

### 2.5. Stability of M. bovirhinis HAZ141_2 Prophage as Well as aadE*-sat4-aphA-3 Gene Cluster under Non-Selective Conditions

Based on the PCR identification of the aadE*-*sat4*-*aphA-3* excision, we investigated whether *M. bovirhinis* HAZ141_2 prophage as well as aadE*-*sat4*-*aphA-3* gene cluster are stable under long-time passages in non-selective conditions (in this study, without Kn) as described in Materials and Methods. The loss of the aadE*-*sat4*-*aphA-3* gene cluster as well as the prophage was monitored selectively in several numbers of culture passages by PCR (semi-quantitate or regular PCR, respectively) allowing detection of the restored sequence junction obtained after excision of either the aadE*-*sat4*-*aphA-3* gene cluster or prophage (Figure S6). The amount of PCR amplification products did not reflect a putative increase of the *M. bovirhinis* HAZ141_2 subpopulation deleted from the aadE*-*sat4*-*aphA-3* cluster (Figure S6A), while loss of the prophage was never detected during 100 passages (Figure S6B).

## 3. Discussion

DNA inversions are known as one of the important motors of chromosome structural changes affecting genotypic and phenotypic diversity, phase variation, and adaptation traits of many bacterial species including *Mycoplasma* [18,19,20]. Results of our study showed that under standard in vitro growth conditions the aadE*-*sat4*-*aphA-3* containing region of the *M. bovirhinis* HAZ141_2 prophage-like element undergoes multiple spontaneous inversions (Figure 1). Two inversion events—through 197-bp-IRs as they exist in the sequenced genome of the *M. bovirhinis* HAZ141-2, and through 155-bp-IRs, obtained from 155-bp DRs established in a genome upon 197-bp-IRs inversion, were confirmed (Figure 1). We hypothesize that inversions, detected in this study, occurred by homologous recombination and are likely driven by a tandem of two serine site-specific DNA recombinases/invertases encoded by the prophage genes located immediately upstream of aadE* (Figure 1; [1,3]). These two Pin-related (COG1961) invertase genes are probably transcribed as an operon, as their coding sequences overlap by 8 nt. A similar arrangement (“in tandem”) of two genes encoding homological site-specific DNA recombinases (nearly 80% identity to that described in this study) was also found in the *Siphoviridae* family viral isolate ctPi910 (BK036829.1) identified from human metagenome [21]. Moreover, many proteins of *M. bovirhinis* HAZ141_2 prophage-like element, related to phage biology, uncharacterized hypothetical proteins, and others (MBVR141_0934-1035) share significant homology (up to 100%) with their counterparts in viral isolate ctPi910 or in other *Siphoviridae* viruses (data not shown), pointing to their possible origin.

Several consequences of the inversions that occurred within the aadE*-*sat4*-*aphA-3* region have been evident. For example, an initial inversion causes a fusion of the MBVR141_0922 to the MBVR141_0926 and an extension of the N-terminal part of the latter by 106 aa at least (Figure S2). The MBVR141_0926 protein is related to DNA-directed RNA polymerase alternative sigma factors, which have key role in the initiation of expression of specialized genes necessary for coping with stress, development, bacterial virulence, etc. [22]. It has been recently shown that *M. genitalium* alternative sigma factor, MG428, is a positive regulator of recombination impacting antigenic variation of *mgp* genes and thus may contribute to the *M. genitalium* virulence [23,24]. Since a function of the MBVR141_0926 protein was out of the scope of this study, we do not know if there is any impact of an N-terminal extension of this protein as well as a 60 aa extension of its C-terminal end obtained as a result of SNP (‘converting’ TAA to TGA), identified in *M. bovirhinis* HAZ141_2 strain present in our laboratory (Figure S2). More studies should be performed to understand the function of MBVR141_0926 in *M. bovirhinis* HAZ141_2 and whether extensions of its both termini influence structural regulation (e.g., via folding of a polypeptide).

The next inversion via 155-bp-IRs results in the changes within the P* promoter region (Figure 1 and Figure 2), located upstream of aadE* gene and supporting co-transcription of the whole aadE*-*sat4*-*aphA-3* gene cluster [3]. We found that the P* sequence was unique (up to September 2021) to *M. bovirhinis* HAZ141_2, while 100% identical sequences to P*^Der^ were identified on certain plasmids of *C. coli* and *C. jejuni* deposited on the NCBI database; moreover, these sequences also included the substitution of ATG start codon of the almost identical aadE* gene for ATA (Figure S3). It is well-known that variations in the promoter region may function as a biological switch to regulate gene expression. For example, inversion and position of the promoter in the “correct” or “incorrect” orientation regulates transcription of Type 1 fimbriae (pili) of *E. coli* [25], or capsular polysaccharides of *Bacteroides fragilis* [26], while the juxtaposition of the active promoter to a silent gene allowed transcription initiation of the recipient gene in mycoplasmas *M. bovis* (*vsp*; [27]) or *M. pulmonis* (*vsa*; [28] as well as *hsd* [29]). In *M. penetrans*, the expression of *mpl* genes encoding P35 related surface membrane lipoproteins depends on the presence of a hairpin structure created by 16-bp IRs located upstream of the promoter-like sequences. This hairpin likely acts as a transcriptional terminator. In the ON promoter configuration, inversion of the promoter-containing region destroys this hairpin and results in the expression of the *mpl* gene [30]. Based on these and other examples existing in the literature, we hypothesized that a different P* promoter sequence (because of inversion) may impact the expression of the aadE*-*sat4*-*aphA-3* genes. However, the results of the antibiotics susceptibility trials did not support our hypothesis and did not reveal any differences in MIC values to Kn/Nm and only a two-fold decrease in the MIC to NTC between pACYC_P* and pACYC_P*^Der^ plasmids (Table 1). Several explanations can be suggested for this finding. First, the plasmid constructs were tested in a Gram-negative *E. coli*, but not in Gram-positive heterological host. However, results of the previous study revealed that the *aphA-3* gene, originated from Gram + as well as Gram- bacteria and cloned under different promoters, was expressed similar in *E. coli* as well as in *Bacillus subtilis* although an additional modification was performed to stabilize the plasmids in *B. subtilis* [11]. Second, even though there are multiple nucleotide differences between P* and P*^Der^, specifically within ‘spacers’ separating -35 and -10 promoter elements as well as two substitutions within -35, the sequences of other promoter-related features such as an extended -10 and TSS remained identical between the two promoters (Figure 2 and Figure S3). Third, previously, co-expression of the aadE*-*sat4*-*aphA-3* genes from P* has been demonstrated [3]. Perhaps the changes within the promoter region are more critical for aadE* expression, to a lesser extent for *sat4* expression, for which a two-fold decrease in MIC to NTC was identified but does not impact the *aphA-3* expression that is mostly dependent on both the ‘internal’ P1″ and P2 promoters (Table 1). The *aadE* gene encodes to 6′ adenyltransferase [AAD(6′)], N-acetyltransferase conferring resistance to Sm [5]. In *M. bovirhinis* HAZ141_2 as well as in certain pathogenic *Campylobacter* isolates, the aadE* gene encodes an N-terminal truncated protein, whose deduced aa sequence contains 228 residues instead of 302 aa of an enzymatically active AadE [3]. No impact of the ‘N-truncated’ AadE* on the MIC to Sm has been observed either in *M. bovirhinis* HAZ141_2 [3] or among the constructs used in this study (data not shown). The finding of aadE* under P*^Der^ in other bacteria and uniqueness of aadE* under P* in *M. bovirhinis* HAZ141_2 (Figure S3) is intriguing and may imply that AadE* might have another unknown enzyme activity different from that of streptomycin adenylyltransferase.

Even though there was no difference in susceptibility to Kn/Nm between *E. coli* cells carrying either pACYC_P* or pACYC_P*^Der^ plasmids, we did show that *aphA-3* gene, expressed solely under its P2 promoter, demonstrated a 4-8 fold decrease in MIC values to Kn/Nm in comparison to the constructs, where *aphA-3* was expressed either under all of three promoters (P*/P*^Der^, P1″, and P2) or under P1″ promoter; the latter located within the *sat4* gene (Table 1 and Table S2, Figure S5). These data are consistent with the previous reports showing that transcription of *aphA-3* was significantly weaker from P2 promoter than from P1 or P1′ (orthologous to P” in our study) in plasmids pJHl (*Enterococcus faecalis* JH1) and pIP1433 (*C. coli* BM2509) as well as in transposon Tn*1545* (*Streptococcus pneumoniae*) [11]. The impact of P* /P*^Der^ on *aphA-3* expression is likely not significant since no difference in the MICs was identified between *aphA-3* expressed simultaneously from three promoters (pACYC_P* and pACYC_P*^Der^) in comparison to *aphA-3* expressed from P1″ alone (pACYC_P1″_∆P2_aphA3; Table 1).

Although, the prophage excision was never identified under conditions used in this study (Figure S6), the PCR analysis did reveal extracellular forms of the aadE*-*sat4*-*aphA-3* gene region (Figure 3C) because of recombination either between the flanking 155 bp DRs (genetic configuration found during the genome sequencing of the *M. bovirhinis* strain HAZ141_2) or between 197 bp DRs (Figure 3A,B). However, we failed either to obtain a Kn-susceptible *M. bovrhinis* HAZ141_2 clone or to show increase of PCR product amount, available upon excision of the aadE*-*sat4*-*aphA-3* cluster, during nonselective serial passages (Figure S6). Probably, the rate of the aadE*-*sat4*-*aphA-3* spontaneous excision is very low in this system and/or under conditions tested in this study, so, only using a high-throughput scanning system could allow identification of Kn-susceptible *M. bovrhinis* HAZ141_2 clones. Recently, nanopore single-molecule long-range technology was used to study stability and dynamics of colistin resistance gene *mcr-1*-bearing transposon Tn*633*0, encoded simultaneously on both plasmid and the chromosome of *E. coli* [31]. The Tn*6330* was very stable in both the plasmid and chromosome after 30 passages without colistin selective pressure and only two single-molecule long reads were found to contain the excised Tn*6330*. The loss of antimicrobial-resistance genes (ARG) after multiple passages without exposure to antibiotic is well-known phenomenon and usually due to the fitness burden on the bacterial host. In contrast, several studies showed the ability of mobile genetic elements carrying ARG to persist in the absence of antibiotic pressure [31,32,33]. Johnsen et al. discussed that the factors such as “rates of reacquisition, effects of resistance traits on bacterial fitness, linked selection, and segregational stability of resistance determinants” may impact the persistence or loss of acquired antimicrobial resistance in bacterial populations [32]. More research should be undertaken to understand if the aadE*-*sat4*-*aphA-3* containing bacteria are putting themselves at an advantage in competition with susceptible counterparts or whether carriage of aadE*-*sat4*-*aphA-3* or prophage impose no detectible fitness cost on *M. bovrhinis* HAZ141_2 and so remain persistent within the genome of the host even in the absence of antibiotic selective pressure.

## 4. Materials and Methods

### 4.1. Mycoplasma bovirhinis Growth Condition

*M. bovirhinis* strain HAZ141-2, isolated from the nasal discharge of a coughing calf in Japan in 2008 [1], was kindly provided by Eiji Hata (National Institute of Animal Health, National Agriculture and Food Research Organization, Sapporo, Hokkaido, Japan). The isolate was propagated at 37 °C, 5% CO_2_ in modified FF broth or agar media [34].

### 4.2. Genomic DNA Extraction and PCR Amplifications

*M. bovirhinis* genomic DNA was extracted using the DNeasy blood and tissue kit (Qiagen, Hilden, Germany) according to the manufacturer’s instructions. The primers were developed and commercially synthesized (Sigma, Rehovot, Israel) based on the nucleotide sequence of the *M. bovirhinis* strain HAZ141_2 genome (Accession number AP018135.1; [1]). The principal primers used in PCR experiments are listed in Table S1. PCRs were carried out in 50 μL volumes containing 50–100 ng of template DNA, 0.5 μL of Phire Hot Start II DNA Polymerase (Thermo Fisher Scientific, Waltham, MA, USA), x5 Phire reaction buffer, 1 μL of 10 mM dNTPs, and 0.4 μM each primer. PCR amplifications were carried out in a C1000 Touch™ Thermal Cycler (Bio-Rad, Hercules, CA, USA). PCR amplicons were purified using the QIAquick gel extraction kit (Qiagen, Hilden, Germany). Sequencing was performed at Hylab (Rehovot, Israel). PCR amplicons used for cloning were extracted from agarose gels and purified using the QIAquick gel extraction kit (Qiagen, Hilden, Germany).

### 4.3. Enzymes and Antibiotics

Restriction enzymes (*Sph*I and *Hind*III) and T4 ligase were purchased from New England Biolabs (NEB) (Ipswich, MA, USA) and Promega, Inc., (Madison, WI, USA), respectively, and used according to the manufacturer’s recommendations. Antibiotics, chloramphenicol, kanamycin sulfate, and neomycin trisulfate hydrate were purchased from Sigma (Sigma-Aldrich, Rehovot, Israel), and nourseothricin sulfate was purchased from ENCO Diagnostics Ltd. (Petach Tikva, Israel).

### 4.4. Construction of pACYC_∆tet∆P2^vec^∆P4^vec^ Derivate Plasmid Vector

The low-copy-number pACYC184 plasmid vector (4245 bp; gifted by M. Kolot, Tel Aviv University) contains p15 A origin of replication as well as *cat* (Cm^R^) and *tet* genes responsible for chloramphenicol (Cm) and tetracycline (Tet) resistance, respectively, and has six promoters/translational initiation sites ([17]; Figure S4). To avoid a possible impact of the plasmid’s P2 and P4 promoters on the expression of the aadE*-*sat4*-*aphA-3* genes cloned downstream of *cat* gene, we modified a pACYC184 plasmid by deletion of those promoters as well as the *tet* gene (pACYC_∆tet∆P2^vec^∆P4^vec^; Figure S4). To generate a pACYC_∆tet∆P2^vec^∆P4^vec^, assembly PCR was undertaken that allows multiple (in our case two) overlapping DNA fragments to be seamlessly linked. Briefly, two pACYC184 derivate DNA fragments were separately amplificated using IS1_del_For and NEW_HindIII_pACYC184 as well as IS1_del_Rev and pACYC184-R1-SphI pair of primers (Table S1). Since the IS1_del_For and IS1_del_Rev primers have a 21 bp overlap, PCR products obtained were future utilized as templates in the next round of PCR using external NEW_HindIII_pACYC184 and pACYC184-R1-SphI primers (for PCR conditions see Table S1). The final pACYC_∆tet∆P2^vec^∆P4^vec^ clone was sequenced to confirm that no mutations had been incorporated during amplifications and manipulations (Hylab; Rehovot, Israel) (Figure S4).

### 4.5. Construction of the P2-Promoterless aphA-3 Gene (∆P2_aphA3) to Test an Influence of P5 Promoter, Regulating Expression of the Cat Gene in pACYC_∆tet∆P2^vec^∆P4^vec^

Two P2 promoterless clones, ∆P2_aphA3-17, for cloning in direction of the *cat* gene and ∆P2_aphA3-19, for cloning in direction opposite to the *cat* gene (Table S2 and Figure S5), were constructed using woP2_aphA_SphI and aphA3-R1-HindIII and woP2_aphA_HindIII and down_aphA-R3_SphI primers, respectively (Table S1). The resulting PCR fragments were purified, sequenced, cut with *Hind*III and *Sph*I, and cloned into pACYC_∆tet∆P2^vec^∆P4^vec^ derivate plasmid. The recombinant clones were transformed into competent cells of *E. coli* strain JM109 (Promega Inc., Madison, WI, USA). The transformants were plated on Luria-Bertani broth (LB) plates containing chloramphenicol (15 μg/mL). DNA of the recombinant plasmids was isolated using PureLink^®^ Quick Plasmid Miniprep Kit (Invitrogen, Waltham, MA, USA) according to the manufacturer’s instructions. The resulting recombinant plasmids pACYC_∆P2_aphA3-17 and pACYC_∆P2_aphA3-19 were completely sequenced (Hylab; Rehovot, Israel) and their susceptibility to Kn and Nm was tested as described below.

### 4.6. Construction of the aadE*-sat4-aphA-3 Gene Cluster Derivatives under Different Promoters

To experimentally validate the expression of the aadE*-*sat4*-*aphA-3* gene cluster derivatives from different promoters, several constructs were prepared (Figure S5) and cloned into pACYC_∆tet∆P2^vec^∆P4^vec^ derivate plasmid (Figure S4) in the direction opposite to the *cat* gene. The list of the recombinant plasmids as well as their description are given in Table S2. While pACYC_P*, pACYC_P*^Der^ and pACYC_P2_aphA3 products were amplified by simple PCR, the pACYC_P* _∆aadE* and pACYC_P1″_∆P2_aphA3 were prepared by assembling PCRs using overlapping primers as described in Table S1. The recombinant clones were transformed into competent cells of *E. coli* strain JM109 (Promega Inc., Madison, WI, USA) as described above and the resulting recombinant plasmids were sequenced (Hylab; Rehovot, Israel). The expression of the aadE*-*sat4*-*aphA-3* gene cluster derivatives from different promoters was assessed by determination of the MICs to Kn/Nm and NTC as described below.

### 4.7. MIC Experiments

The in vitro susceptibility of *E. coli* strain JM109 and that of its transformants carrying either the empty pACYC184 vector or the recombinant clones was tested using the agar dilution method following instructions of the European Committee for Antimicrobial Susceptibility Testing (EUCAST) of the European Society of Clinical Microbiology and Infectious Diseases (ESCMID) [35] as previously described [3].

### 4.8. Serial Passage of M. bovirhinis Strain HAZ141-2 under Nonselective Conditions

*M. bovirhinis* strain HAZ141-2 broth culture was grown for 16–24 h until color change in modified FF medium as described above. Then, the culture was diluted 1:100 in FF to start the subsequent cycle. Passaging was repeated for 100 times, and presence of *M. bovirhinis* population negative either for aadE*-*sat4*-*aphA-3* gene cluster or for the prophage was determined every 20–30th passages using semi-quantitative PCR or regular PCR, respectively, both allowing detection of the restored sequence junction obtained after aadE*-*sat4*-*aphA-3* excision or prophage excision (Figure S6 and Table S1).

### 4.9. Computational Analysis

BLAST analysis of protein and nucleotide sequences was performed using the NCBI server (https://blast.ncbi.nlm.nih.gov/Blast.cgi (accessed on 29 September 2021)). The different functional domains were identified using the Pfam protein family’s database (http://pfam.xfam.org/ (accessed on 29 September 2021)), integrated resource of Protein Domains (InterPro) (https://www.ebi.ac.uk/interpro/ (accessed on 29 September 2021)), and the database of protein families and domains PROSITE (https://prosite.expasy.org/ (accessed on 29 September 2021)). Primary DNA sequence analyses (GC content, direct repeats, dyade symmetries etc.) were performed either with the Clone Manager 9 Professional Edition software (Scientific & Educational Software, Durham, NC, USA), or DNASTAR software, version 5.06/5.51, 2003 (Lasergene Inc., Madison, WI, USA). DNA promoter motif searches were performed with the Pattern Locator program [36] (https://www.cmbl.uga.edu/software/patloc.html (accessed on 29 September 2021)).

## 5. Conclusions

This study shows that the *aadE*-*sat4*-*aphA-3* gene cluster can undergo inversions and deletions in mycoplasma, which do not affect a stable inheritance of the above genes in the bacterial population, at least at the conditions tested in this study. Perhaps, for some yet unknown reason, the presence and the expression of the aadE*-*sat4*-*aphA-3* genes are important for prophage or/and *M. bovirhinis* HAZ141_2 itself, and because of that, the “bacterium/prophage system” does not allow hampering of the above genes during rearrangements. Indirect proof for the importance of the aadE*-*sat4*-*aphA-3* gene cluster as well as prophage for *M. bovirhinis* HAZ141_2 may be found from the fact that we failed to obtain either aadE*-*sat4*-*aphA-3* or prophage ‘cured’ clones during multiple passages under non-selective conditions. There is also a possibility that the inversion(s), detected by us in vitro, does not occur in vivo since they do not bring an advantage or a functional basis for the host. More studies should be performed to understand if aadE*-*sat4*-*aphA-3* gene cluster as well as prophage may impact *M. bovirhinis* HAZ141_2 fitness. 

## Figures and Tables

**Table 1:** In vitro susceptibility to aminoglycosides and nourseothricin of *E. coli* JM109 clones carrying recombinant plasmids containing different derivatives of the *M. bovirhinis* HAZ141_2 aadE*-*sat4*-*aphA-3* gene cluster.

Plasmids	MIC (μg/mL)		
	Kn	Nm	NTC
pACYC184	1	1	1
pACYC_P*	512–1024	1024	512
pACYC_P*^Der^	512–1024	1024	256
pACYC_P* _∆aadE*	512–1024	1024	512
pACYC_P1″_∆P2_aphA3	512–1024	1024	NA
pACYC_P2_aphA3	128	128	NA
pACYC_∆P2_aphA3-17	256–512	256–512	NA
pACYC_∆P2_aphA3-19	4	4	NA

Kn, kanamycin; Nm, neomycin; NTC, nourseothricin. NA—not applicable.

## Data Availability

The data presented in this study are available on request from the corresponding authors. The data supporting the findings of this study are available within the paper and its supplemental material.

## Supplementary Materials

The following are available online at https://www.mdpi.com/article/10.3390/antibiotics10111335/s1, Figure S1: Two types of repeated DNA sequences of 155 and 197 base pairs (bps) each, which flank and overlap with the primary promoter P* located upstream of the *aadE**, Figure S2: Amino acid sequence alignment of *M. bovirhinis* strain HAZ141_2 putative DNA-directed RNA polymerase alternative sigma factor (MBVR141_0926) before and after inversion through 197-bp long inverted repeats, Figure S3: The closest homologous sequences of the putative promoters located upstream of *aadE**- and *aadE*-like genes in genomes of other bacteria, Figure S4: Sequence of the pACYC_∆tet∆P2^vec^∆P4^vec^ cloning vector used in this study, Figure S5: Schematics (as circular maps) of the seven plasmid constructs used in this study and nucleotide sequences of the *aadE**-*sat4*-*aphA-3* derivatives used for cloning into unique *Hind*III and *Sph*I restriction sites of the pACYC_∆tet∆P2^vec^∆P4^vec^ vector (Figure S4), Figure S6: Analyses of a potential loss of the *aadE**-*sat4*-*aphA-3* gene cluster as well as a prophage during multiple passages of *M. bovirhinis* HAZ141_2 under nonselective conditions, Table S1: Oligonucleotides used in this study, Table S2: Description of the recombinant plasmids used in this study.
