# Supplementary material for: The aadE*-sat4-aphA-3 Gene Cluster of Mycoplasma bovirhinis HAZ141_2 Undergoes Genomic Rearrangements Influencing the Primary Promoter Sequence
Source: Antibiotics (Basel). 2021 Nov 1;10(11):1335. doi: 10.3390/antibiotics10111335 (PMC8614714; doi:10.3390/antibiotics10111335)
Supplement: Supplementary file 1 [file antibiotics-10-01335-s001.zip › antibiotics-1428173-supplementary/Fig_S1-S2.pdf]

antibiotics

Figure S1

>197 bp Inverted Repeat Left (IRL; nc 870715-870911; MBVR141_0922)
ATTTTTTCTT**t**TACTGC**t**GAAACAGTATCTTTGATTGAATGCGTAGTTGTATCTATAACATTTAA
TTCATAGATTCC**t**AAATTGGAAAATTG**a**TTCCACATTGTTTCAACTAATTCAATATTTGTTTCTC
TGTCTAACTTTGAGCGTTCGATAGCTCGCTTCATAGTTTCTTCTTTACTTGCCCTTAAAACGATA
TA

>197 bp Inverted Repeat Right (IRR; nc 873765-873961; MBOV141_0926)
TATATCGTTTTAAGGGCAAGTAAAGAAGAAACTATGAAGCGAGCTATCGAACGCTCAAAGTTAGA
CAGAGAAACAAATATTGAATTAGTTGAAACAATGTGGAA**g**CAATTTTCCAATTT**g**GGAATCTATG
AATTAAATGTTATAGATACAACTACGCATTCAATCAAAGATACTGTTTC**c**GCAGTA**c**AAGAAAAA
AT

>155 bp Direct Repeat Left (DRL; nc 871101-871255; MBOV141_0922)
ATAATGATAAAAGTCATCTGTGTGCATATGCACAGACTTTTCCAAATCTGATTCTTTTGCAACAG
CAGATGCCGTTGTAGTTTTTCCTGTCCCCGGCGCACCTGTGATTACAATAATTCTACCTTGATT**t**
ATCTATATTTTACCTCC**a**CAAATTC

>155 bp Direct Repeat Right (DRR; (876931-877085; MBOV141_0932)
ATAATGATAAAAGTCATCTGTGTGCATATGCACAGACTTTTCCAAATCTGATTCTTTTGCAACAG
CAGATGCCGTTGTAGTTTTTCCTGTCCCCGGCGCACCTGTGATTACAATAATTCTACCTTGATT**c**
ATCTATATTTTACCTCC**g**CAAATTC

Figure S1: Two types of repeated DNA sequences of 155 and 197 base pairs (bps) each, which flank and overlap with the primary promoter P* located upstream of the *aadE**. All numbers of nucleotide coordinates are according to the *M. bovirhinis* strain HAZ141_2 genome (accession number AP018135.1; [1]). Mismatches between two 197 bp IRs and those between two 155 bp DRs are shown as lower-case bolded and underlined letters.

References

1. Hata, E.; Nagai, K.; Murakami, K. Complete genome sequence of *Mycoplasma bovirhinis* strain HAZ141_2 from bovine nasal discharge in Japan. *Genome Announc*. **2017,** *5*:e01000-01017.

```
Sigma-70 factor          --------------------------------------------------
MBVR141_0926_162         --------------------------------------------------
MBVR141_0926_222         --------------------------------------------------
MBVR141_0926_328         MNQGRIIVITGAPGTGKTTTASAVAKESDLEKSVHMHTDDFYHYLSKGAI
AAA family ATPase        MNQGRIIVITGAPGTGKTTTASAVAKESDLEKSVHMHTDDFYHYLSKGAI
Sigma-70 factor          --------------------------------------------------
MBVR141_0926_162         --------------------------------------------------
MBVR141_0926_222         --------------------------------------------------
MBVR141_0926_328         PPHLPESNEQNLIVIEAFLEAAKRYARGGYDVIVDGIVGPWFLEPWLNIV
AAA family ATPase        PPHLPESNEQNLIVIEAFLEAAKRYARGGYDVIVDGIVGPWFLEPWLNIV

Sigma-70 factor          ------MYIVLRASKEETMKRAIERSKLDRETNIELVETMWKQFSNLGIY
MBVR141_0926_162         ------MYIVLRASKEETMKRAIERSKLDRETNIELVETMWKQFSNLGIY
MBVR141_0926_222         ------MYIVLRASKEETMKRAIERSKLDRETNIELVETMWKQFSNLGIY
MBVR141_0926_328         QEHYEVHYIVLRASKEETMKRAIERSKLDRETNIELVETMWKQFSNLGIY
AAA family ATPase        QEHYEVMYIVLRASKEETMKRAIERSKLDRETNIELVETMWKQFSNLGIY
                               ********************************************

Sigma-70 factor          ELNVIDTTTHSIKDTVSAVQEKIASKRTTLVEESELTHVAAPDNPTKEFE
MBVR141_0926_162         ELNVIDTTTHSIKDTVSAVQEKIASKRTTLVEESELTHVAAPDNPTKEFE
MBVR141_0926_222         ELNVIDTTTHSIKDTVSAVQEKIASKRTTLVEESELTHVAAPDNPTKEFE
MBVR141_0926_328         ELNVIDTTTHSIKDTVSAVQEKIASKRTTLVEESELTHVAAPDNPTKEFE
AAA family ATPase        ELNVIDTTTHSIKDTVSAVKEKIVSG-TALLF------------------
                         *******************:***.*  *:*:

Sigma-70 factor          NRQERFALYRAMQQLDAETREVIHLRLAGDFSFRDIGDILGHSEVWARVR
MBVR141_0926_162         NRQERFALYRAMQQLDAETREVIHLRLAGDFSFRDIGDILGHSEVWARVR
MBVR141_0926_222         NRQERFALYRAMQQLDAETREVIHLRLAGDFSFRDIGDILGHSEVWARVR
MBVR141_0926_328         NRQERFALYRAMQQLDAETREVIHLRLAGDFSFRDIGDILGHSEVWARVR
AAA family ATPase        --------------------------------------------------

Sigma-70 factor          FYRGKEKLVKIIGGDNNA--------------------------------
MBVR141_0926_162         FYRGKEKLVKIIGGDNNA--------------------------------
MBVR141_0926_222         FYRGKEKLVKIIGGDNNAWNQSWLLRSQGFTPILSWRIDRDRDLCYGQRT
MBVR141_0926_328         FYRGKEKLVKIIGGDNNAWNQSWLLRSQGFTPILSWRIDRDRDLCYGQRT
AAA family ATPase        --------------------------------------------------

Sigma-70 factor          ----------------------------
MBVR141_0926_162         ----------------------------
MBVR141_0926_222         HWALCGLPPAWRGYAQAGSYWKSATACT
MBVR141_0926_328         HWALCGLPPAWRGYAQAGSYWKSATACT
AAA family ATPase        ----------------------------
```

Figure S2: amino acid sequence alignment of *M. bovirhinis* strain HAZ141_2 putative DNA-directed RNA polymerase alternative sigma factor (MBVR141_0926) before and after inversion through 197-bp long inverted repeats. The comparison includes three derivative variants of *M. bovirhinis* MBVR141_0926 protein: (i) 162 aa polypeptide (as found during the genome sequencing of the *M. bovirhinis* strain HAZ141_2; accession no. BBA22581.1 [1]); (ii) 222 aa polypeptide because of a single-nucleotide polymorphism within the annotated stop codon TAA converting it to TGA encoding tryptophan in mycoplasmas (as found in *M. bovirhinis* strain HAZ141_2 present in our laboratory) and (iii) 328 aa polypeptide received as a result of the inversion through 197-bp long inverted repeats (Fig. 1A-B). A 106 aa N-terminal extension, appeared in the 328 aa protein is shown in red, while a 60 aa C-terminal extension, appeared in

222 and in 328 aa proteins is shown in blue. In addition, the AAA family ATPase (MULTISPECIES [Bacteria]; RefSeq: WP_039121379.1) and sigma-70 family RNA polymerase sigma factor (MULTISPECIES [Bacteria]; RefSeq: WP_197713445.1) were added to the comparison. The AAA-ATPases domain (7-140 aa; pfam13238), identified in the long 328 aa protein is shaded by grey, while the RNA polymerase sigma-70 factor family domain (173-258 aa; TIGR02937), found in all variants of the MBVR141_0926 protein, is shaded by blue-green. Alignment length is 328 aa residues; the protein sequences were aligned using the CLUSTAL W public server (https://npsa-prabi.ibcp.fr/cgi-bin/npsa_automat.pl?page=/NPSA/npsa_clustalwan.html) [2]. Identical aa are marked with an asterisk, strongly aa with a colon, while weakly similar aa with a dot. The 1st N-terminal residues are underlined as follow: in the short versions the predicted start codon for translation of MBVR141_0926 is TTG (formally encodes Leu), while in the extended version it is ATA (formally encodes Ile). Both the Leu and Ile are known coding for fMet and used as alternate start codons [3-4].

References

1. Hata, E.; Nagai, K.; Murakami, K. Complete genome sequence of *Mycoplasma bovirhinis* strain HAZ141_2 from bovine nasal discharge in Japan. *Genome Announc.* **2017**, *5*:e01000-01017.
2. Thompson, J.D.; Higgins, D.G.; Gibson, T.J. CLUSTAL W: improving the sensitivity of progressive multiple sequence alignment through sequence weighting, position-specific gap penalties and weight matrix choice. *Nucleic Acids Res.* **1994**, *22*, 4673-4680.
3. Hecht, A.; Glasgow, J.; Jaschke, P. R.; Bawazer, L. A.; Munson, M. S.; Cochran, J. R.; Endy, D.; Salit, M. Measurements of translation initiation from all 64 codons in *E. coli*. *Nucleic Acids Res.* **2017,** *45*, 3615-3626.
4. Chang, C.-P.; Chen, S.-J.; Lin, C.-H.; Wang, T.-L.; Wang, C.-C. A single sequence context cannot satisfy all non-AUG initiator codons in yeast. *BMC Microbiol.* **2010,** *10*, 188-196.
