# Supplementary material for: The aadE*-sat4-aphA-3 Gene Cluster of Mycoplasma bovirhinis HAZ141_2 Undergoes Genomic Rearrangements Influencing the Primary Promoter Sequence
Source: Antibiotics (Basel). 2021 Nov 1;10(11):1335. doi: 10.3390/antibiotics10111335 (PMC8614714; doi:10.3390/antibiotics10111335)
Supplement: Supplementary file 1 [file antibiotics-10-01335-s001.zip › antibiotics-1428173-supplementary/Fig_S3-S6.pdf]

antibiotics MDPI

```
                                -35                ext.-10      +1                  RBS         fMet
M. bovirh.HAZ141_2_P*      TTGAAA-AAATGTAAAATTTGTGATATAAT-AAACTGAACGATAAATAAGAATTTGCGGAGGTAAAATATAGATG aadE* →
M. bovirh.HAZ141_2_P*Der   ATGATACAGTGAATAGATTTATGATATAAT-GAG-TTATCAACAAATCGGAATTTGTGGAGGTAAAATATAGATA aadE* →
                           ****************************** *** ****************************************

C. coli pCFSAN032805       ATGATACAGTGAATAGATTTATGATATAAT-GAG-TTATCAACAAATCGGAATTTGTGGAGGTAAAATATAGATA aadE* →
C. coli pCFSAN032806       ATGATACAGTGAATAGATTTATGATATAAT-GAG-TTATCAACAAATCGGAATTTGTGGAGGTAAAATATAGATA aadE* →
C. coli pCCDM183           ATGATACAGTGAATAGATTTATGATATAAT-GAG-TTATCAACAAATCGGAATTTGTGGAGGTAAAATATAGATA aadE* →
C. jejuni pGMI16-002       ATGATACAGTGAATAGATTTATGATATAAT-GAG-TTATCAACAAATCGGAATTTGTGGAGGTAAAATATAGATA aadE* →

C. jejuni pCG8245          TTGAAA-AGTGGATAGATTTATGATACAGT-GAATAGA..+29 bp..GGAATTTGTGGAGGTAAAATATAGATA aadE* →
C. jejuni pGB19            GTGTTT-CTTATGCTGTTGTATGGTATgAT-TAGTAAG..+20 bp..AGAATTTGTGGAGGTAAAATATAGATG aadE* →

E. faecalis plasmid        TTGTATCGGCAAAAAAGAAGATGTAATAAT-ATAGAAA...........AAATAAAC-GAGG-AGTATGTAAATG aadE  →
S. aureus Tn5405           TTGTATCGGCAAAAAAGAAGATGTAATAAT-ATAGAAA...........AAATAAAC-GAGG-AGTATGTAAATG aadE  →
S. epidermidis plasmid     TTGTATCGGCAAAAAAGAAGATGTAATAAT-ATAGAAA...........AAATAAAC-GAGG-AGTATGTAAATG aadE  →

S. pyogenes NGAS322        TTTTAA-AATAGTAGACTATGTGATATAAT-AGAATCG...........GAAGTTGTGGAGGTAAAATATAGATG aadE* →
S. suis PP phi-SsUD.1      TTAAAAAAATCGTTTTATGGTTGTTCAAATTATCCTGAA..+35 bp..AAAATAAACGAGG-AGTATGTAAATG aadE  →
```

Figure S3: the closest homologous sequences of the putative promoters located upstream of *aadE**- and *aadE*-like genes in genomes of other bacteria. Nucleotide sequence alignment of *Mycoplasma bovirhinis* HAZ141_2 P* and P*^{Der} promoters with their closest homologous identified in genomes of other bacteria. The sequences were aligned using the CLUSTALW [1] public server (https://npsa-prabi.ibcp.fr/cgi-bin/npsa_automat.pl?page=/NPSA/npsa_clustalwan.html). Promoter elements, as previously identified for P* [2] are shown with -35 and extended -10 (as ext. -10) above the boldface letters. The 5'RACE experimentally validated transcription start site is shown with +1 above, boldfaced and underlined. Predicted ribosome-binding site is shown with RBS above the boldface italicized letters. Arrow indicates the position and direction of transcription of the *aadE** gene, putative start codons of which (ATG before and ATA after inversion) are shown in bold and underlined. The identical nucleotides identified between *M. bovirhinis* HAZ141_2 P*^{Der} promoter and its homologous sequences in genomes of other bacteria are marked with asterisks.

C. coli pCFSAN032805 - *Campylobacter coli* strain CFSAN032805, plasmid pCFSAN032805 (CP045793.1).

C. coli pCFSAN032806 - *Campylobacter coli* strain CFSAN032806, plasmid pCFSAN032806 (either CP045790.1, or CP023544.1).
C. coli pCCDM183 - *Campylobacter coli* strain BP3183 plasmid pCCDM183 (CP017872.1).
C. jejuni pGMI16-002 - *Campylobacter jejuni* strain CFSAN054107 plasmid pGMI16-002 (CP028186.1).
C. jejuni pCG8245 - *Campylobacter jejuni* strain CG8245, plasmid pCG8245 (AY701528.1).
C. jejuni pGB19 - *Campylobacter jejuni* strain GB19, plasmid pGB19 (CP071593.1).
E. faecalis plasmid - *Enterococcus faecalis* strain OG1RF x UW3114 T-12, plasmid pLG2 (HQ426665.1).
S. aureus Tn5405 – *Staphylococcus aureus* strain, transposon Tn*5405* (U73026).
S. epidermidis plasmid - *Staphylococcus epidermidis* strain RP62A, plasmid pSERP (CP000028.1).
S. pyogenes NGAS322 – *Streptococcus pyogenes* strain NGAS322 (CP010449.1).
S. suis PP phi-SsUD.1 - *Streptococcus suis* strain SsUD, prophage phi-SsUD.1 (FN997652.1).

References:

1. Thompson, J.D.; Higgins, D.G.; Gibson, T.J. CLUSTAL W: improving the sensitivity of progressive multiple sequence alignment through sequence weighting, position-specific gap penalties and weight matrix choice. *Nucleic Acids Res.* **1994,** *22*, 4673-4680.

2. Lysnyansky, I; Borovok, I. A GC-rich prophage-like genomic region of *Mycoplasma bovirhinis* HAZ141_2 carries a gene cluster encoding resistance to kanamycin and neomycin. *Antimicrob Agents Chemother*. **2021,** *65*:e01010-20. doi: 10.1128/AAC.01010-20.

```
    SphI       pACYC184-R1-SphI →
  1 GCATGCGTAT TAACGAAGCG CTAACCGTTT TTATCAGGCT CTGGGAGGCA GAATAAATGA TCATATCGTC AATTATTACC TCCACGGGGA
 91 GAGCCTGAGC AAACTGGCCT CAGGCATTTG AGAAGCACAC GGTCACACTG CTTCCGGTAG TCAATAAACC GGTAAACCAG CAATAGACAT
181 AAGCGGCTAT TTAACGACCC TGCCCTGAAC CGACGACCGG GTCGAATTTG CTTTCGAATT TCTGCCATTC ATCCGCTTAT TATCACTTAT

                +A283
271 TCAGGCGTAG CAACCAGGCG TTTAAGGGCA CCAATAACTG CCTTAAAAAA ATTACGCCCC GCCCTGCCAC TCATCGCAGT ACTGTTGTAA
                                                         <<................cat...................<
220                                                        *  A  G   G  Q  W   E  D  C   Y  Q  Q  L
                                           397
361 TTCATTAAGC ATTCTGCCGA CATGGAAGCC ATCACAAACG GCATGATGAA CCTGAATCGC CAGCGGCATC AGCACCTTGT CGCCTTGCGT
    <.........................................cat..........................................<
207   E  N  L   M  R  G   V  H  F  G   D  C  V   A  H  H   V  Q  I  A   L  P  M   L  V  K   D  G  Q  T

451 ATAATATTTG CCCATGGTGA AAACGGGGGC GAAGAAGTTG TCCATATTGG CCACGTTTAA ATCAAAACTG GTGAAACTCA CCCAGGGATT
    <.........................................cat..........................................<
177   Y  Y  K   G  M  T   F  V  P  A   F  F  N   D  M  N   A  V  N  L   D  F  S   T  F  S   V  W  P  N

541 GGCTGAGACG AAAAACATAT TCTCAATAAA CCCTTTAGGG AAATAGGCCA GGTTTTCACC GTAACACGCC ACATCTTGCG AATATATGTG
    <.........................................cat..........................................<
147   A  S  V   F  F  M   N  E  I  F   G  K  P   F  Y  A   L  N  E  G   Y  C  A   V  D  Q   S  Y  I  H

631 TAGAAACTGC CGGAAATCGT CGTGGTATTC ACTCCAGAGC GATGAAAACG TTTCAGTTTG CTCATGGAAA ACGGTGTAAC AAGGGTGAAC
    <.........................................cat..........................................<
117   L  F  Q   R  F  D   D  H  Y  E   S  W  L   S  S  F   T  E  T  Q   E  H  F   V  T  Y   C  P  H  V

                                                1680
721 ACTATCCCAT ATCACCAGCT CACCGTCTTT CATTGCCATA CGGAATTCCG GATGAGCATT CATCAGGCGG GCAAGAATGT GAATAAAGGC
    <.........................................cat..........................................<
 87   S  D  W   I  V  L   E  G  D  K   M  A  M   R  F  E   P  H  A  N   M  L  R   A  L  I   H  I  F  A

811 CGGATAAAAC TTGTGCTTAT TTTTCTTTAC GGTCTTTAAA AAGGCCGTAA TATCCAGCTG AACGGTCTGG TTATAGGTAC ATTGAGCAAC
    <.........................................cat..........................................<
```

```
57     P  Y  F   K  H  K   N  K  K  V   T  K  L   F  A  T   I  D  L  Q   V  T  Q   N  Y  T   C  Q  A  V

                                                                                                    RBS
901    TGACTGAAAT GCCTCAAAAT GTTCTTTACG ATGCCATTGG GATATATCAA CGGTGGTATA TCCAGTGATT TTTTTCTCCA TTTTAGCTTC
       <........................................cat........................................<<
27     S  Q  F   A  E  F   H  E  K  R   H  W  Q   S  I  D   V  T  T  Y   G  T  I   K  K  E   M

                                                                +1?      ← -10/P5               ← -35/P5
991    CTTAGCTCCT GAAAATCTCG ATAACTCAAA AAATACGCCC GGTAGTGATC TTATTTCATT ATGGTGAAAG TTGGAACCTC TTACGTGCCG

1081   ATCAACGTCT CATTTTCGCC AAAAGTTGGC CCAGGGCTTC CCGGTATCAA CAGGGACACC AGGATTTATT TATTCTGCGA AGTGATCTTC

                         ← IS1_del_Rev          IS1_del_For →
1171   CGTCACAGGT ATTTATTCGG CGCAAAGTGC GTCGGCGGAG TGTATACTGG CTTACTATGT TGGCACTGAT GAGGGTGTCA GTGAAGTGCT

1261   TCATGTGGCA GGAGAAAAAA GGCTGCACCG GTGCGTCAGC AGAATATGTG ATACAGGATA TATTCCGCTT CCTCGCTCAC TGACTCGCTA

1351   CGCTCGGTCG TTCGACTGCG GCGAGCGGAA ATGGCTTACG AACGGGGCGG AGATTTCCTG GAAGATGCCA GGAAGATACT TAACAGGGAA

1441   GTGAGAGGGC CGCGGCAAAG CCGTTTTTCC ATAGGCTCCG CCCCCCTGAC AAGCATCACG AAATCTGACG CTCAAATCAG TGGTGGCGAA
1531   ACCCGACAGG ACTATAAAGA TACCAGGCGT TTCCCCCTGG CGGCTCCCTC GTGCGCTCTC CTGTTCCTGC CTTTCGGTTT ACCGGTGTCA
1621   TTCCGCTGTT ATGGCCGCGT TTGTCTCATT CCACGCCTGA CACTCAGTTC CGGGTAGGCA GTTCGCTCCA AGCTGGACTG TATGCACGAA
1711   CCCCCCGTTC AGTCCGACCG CTGCGCCTTA TCCGGTAACT ATCGTCTTGA GTCCAACCCG GAAAGACATG CAAAAGCACC ACTGGCAGCA
1801   GCCACTGGTA ATTGATTTAG AGGAGTTAGT CTTGAAGTCA TGCGCCGGTT AAGGCTAAAC TGAAAGGACA AGTTTGGTG ACTGCGCTCC
1891   TCCAAGCCAG TTACCTCGGT TCAAAGAGTT GGTAGCTCAG AGAACCTTCG AAAAACCGCC CTGCAAGGCG GTTTTTTCGT TTTCAGAGCA
1981   AGAGATTACG CGCAGACCAA AACGATCTCA AGAAGATCAT CTTATTAATC AGATAAAATA TTTCTAGATT TCAGTGCAAT TTATCTCTTC

                                       ← NEW_HindIII_pACYC184  HindIII
2071   AAATGTAGCA CCTGAAGTCA GCCCCATACG ATATAAGTTG TAATTCTCAT GTAAGCTT
```

Figure S4: sequence of the pACYC_ΔtetΔP2^{vec}ΔP4^{vec} cloning vector used in this study. The 2,128 bp pACYC_ΔtetΔP2^{vec}ΔP4^{vec} cloning vector was constructed from the low-copy-number pACYC184 plasmid (4,245 bp; GenBank X06403.1) by deletion of its P2 and P4 promoters (described

by Stuber & Bujard [1] and designated here as P2^{vec} and P4^{vec}) as well as the *tet* gene as described in Materials and Methods and in Tables S1-2. Names of primers (pACYC184-R1-SphI, IS1_del_For, IS1_del_Rev, and NEW_HindIII_pACYC184) used for assembling PCR shown above the corresponding sequences in bold blue; overlapping sequences of IS1_del_For and IS1_del_Rev are underlined. Both start and stop codons of the *cat* gene (a minus strand) encoding resistance to chloramphenicol shown in bold red and underlined. The *cat*-P5 promoter-related elements including +1 (transcription start site), -10, and -35 elements are bolded and underlined; the predicted ribosome-binding site (RBS) is shown in bold italic. The p15A origin of replication is grey highlighted. One single nucleotide polymorphism (SNP of G to A; a silent mutation) has been identified at position 397 of the pACYC_ΔtetΔP2^{vec}ΔP4^{vec} within the encoding sequence of the *cat* gene results in silent mutation. In addition, an indel (as insertion) of a nucleotide "A" (+A283) was found at position 283 of the pACYC_ΔtetΔP2^{vec}ΔP4^{vec} located downstream of the *cat* gene. The newly designed restriction sites *Hind*III and *Sph*I, used for the cloning in this study, are italicized, bolded and underlined. The nucleotide 1680 (in bold and green highlighted) corresponds to the 1st nucleotide of pACYC184 (X06403.1).

References

1. Stuber, D.; Bujard, H. Organization of transcriptional signals in plasmids pBR322 and pACYC184. *Proc Natl Acad Sci U S A* **1981,** *78*, 167-71.

Figure S5: schematics (as circular maps) of the seven plasmid constructs used in this study and nucleotide sequences of the *aadE**-*sat4*-*aphA-3* derivatives used for cloning into unique *Hind*III and *Sph*I restriction sites of the pACYC_ΔtetΔP2^{vec}ΔP4^{vec} vector (Fig. S4).

All of schematics contain the following common elements: genes shown as bended arrows colored with yellow for *cat* (resistance to chloramphenicol), red for *aadE**, blue for *sat4*, and green for *aphA-3*; promoters shown as colored rectangles, whose colors correspond to their cognate genes (e.g. P5 of *cat* is yellow, P2 of *aphA-3* is green etc). The plasmid p15A origin of replication shown as a grey bended rectangle and signed as *ori* p15A. Black rectangles mark positions of predicted transcriptional terminators signed with T capital. Each of schematics is followed with a nucleotide sequence of the corresponding cloned insert. Open reading frames (ORFs) of *aadE**, *sat4* and *aphA-3* are shown in bold and colored as described above – red for *aadE**, blue for *sat4*, and green for *aphA-3* except a four-nucleotide overlapping between the stop codon of *aadE** (TGA) and the start of *sat4* (GTG), which colorless and underlined. An ORF remnant (if there is) shown with same color as that of an intact ORF. Start and stop codons of ORFs underlined. Promoter sequences shown in bold and highlighted using the following colors – grey for P* and P*^{Der}, yellow for P1'', and green for P2.

pACYC_P* (4,343 bp)

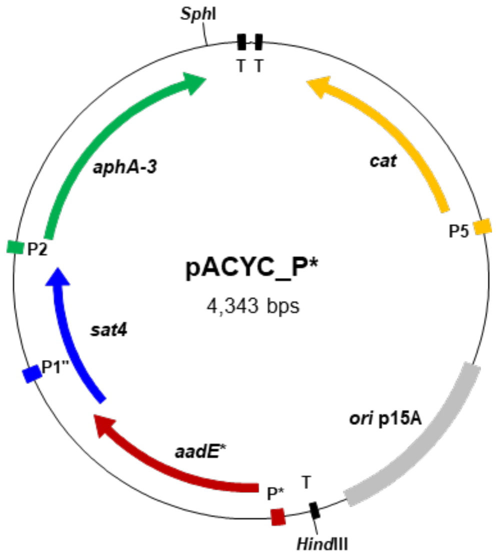

pACYC_P* (an insert size is 2,215 bp; without flanking *Hind*III and *Sph*I sites)

*Hind*III

AAGCTTGCACATTCGGTAACGGAAGCAGTTACAATCTCCTGCAAAGTCGGGAGATTTTTTCGTTATACGGTAGGTGAATG
TA**TTGAAAAAATGTAAAATTTGTGATATAAT**AAACTGAACGATAAATAAGAATTTGCGGAGGTAAAATATAG**ATGAATCA**
AGGTAGAATTATTGTAATCACAGGTGCGCCGGGGACAGGAAAAACTACAACGGCATCTGCTGTTGCAAAAGAATCAGATT
TGGAAAAGTCTGTGCATATGCACACAGATGACTTTTATCATTATCATGTAAGAAAGCCAAGCGCAAGGGAGTATGATGAT
TGCTGCAATGAATTTTGGAATGTAACAGCTTATGTTATTAAAGGATTGTGCCGTAAGGAAATTTTATTTGCTATTGATCA
TTTTAATCAGATTGTTCGCCATGAGCTGCTGAGAATGATATCATGGAAGGTCGGCATCGAAACAGGCTTTAAATTAAGTG
TAGGCAAGAACTATAAGTTTATTGAAAGGTATGTATCCGAGGATTTGTGGGAGAAACTTTTGTCCACCTACCGGATGGAT
TCCTATGAAAACATATGGGAAGCATTATTTCTATGCCATCAATTGTTCAGGGCGGTATCCGGTGAGGTGGCGGAATGGCT
TCATTATGCCTATCCGGAGTATGATAGGAATATAACAAAATATACCAGGGACATGTATAAAAAATACACTGGTAAAACCG
GCTGCCTGGATAGCACATATGCCGCTGATATAGAAGAGAGGCGGGAACAGTGATTACAGAAATGAAAGCAGGGCACCTGA
AAGATATCGATAAACCCAGCGAACCATTTGAGGTGATAGGTAAGATTATACCGAGGTATGAAAACGAGAATTGGACCTTT
ACAGAATTACTCTATGAAGCGCCATATTTAAAAAGCTACCAAGACGAAGAGGATGAAGAGGATGAGGAGGCAGATTGCCT
TGAATATATTGACAATACTGATAAGATAATATATCTTTACTACCAAGACGATAAATGCGTCGGAAAAGTTAAACTGCGAA
AAAATTGGAACCGGTACGCTTATATAGAAGATATCGCCGTATGTAAGGATTTCAGGGGGCAAGGCATAGGCAGCGCGCTT
ATCAATATATCTATAGAATGGGCAAAGCATAAAAACTTGCATGGACTAATGCTTGAAACCCAGGACAATAACCTTATAGC
TTGTAAATTCTATCATAATTGTGGTTTCAAAATCGGCTCCGTCGATACTATGTTATACGCCAACTTTGAAAACAACTTTG
AAAAAGCTGTTTTCTGGTATTTAAGGTTTTAGAATGCAAGGAACAGTGAATTGGAGTTCGTCTTGTTATAATTAGCTTCT
TGGGGTATCTTTAAATACTGTAGAAAAGAGGAAGGAAATAATAA**ATGGCTAAAATGAGAATATCACCGGAATTGAAAAAA**
CTGATCGAAAAATACCGCTGCGTAAAAGATACGGAAGGAATGTCTCCTGCTAAGGTATATAAGCTGGTGGAGAAAATGA
AAACCTATATTTAAAAATGACGGACAGCCGGTATAAAGGGACCACCTATGATGTGGAACGGGAAAAGGACATGATGCTAT
GGCTGGAAGGAAAGCTGCCTGTTCCAAAGGTCCTGCACTTTGAACGGCATGATGGCTGGAGCAATCTGCTCATGAGTGAG
GCCGATGGCGTCCTTTGCTCGGAAGAGTATGAAGATGAACAAAGCCCTGAAAAGATTATCGAGCTGTATGCGGAGTGCAT
CAGGCTCTTTCACTCCATCGACATATCGGATTGTCCCTATACGAATAGCTTAGACAGCCGCTTAGCCGAATTGGATTACT
TACTGAATAACGATCTGGCCGATGTGGATTGCGAAAACTGGGAAGAAGACACTCCATTTAAAGATCCGCGCGAGCTGTAT
GATTTTTTAAAGACGGAAAAGCCCGAAGAGGAACTTGTCTTTTCCCACGGCGACCTGGGAGACAGCAACATCTTTGTGAA
AGATGGCAAAGTAAGTGGCTTTATTGATCTTGGGAGAAGCGGCAGGGCGGACAAGTGGTATGACATTGCCTTCTGCGTCC

GGTCGATCAGGGAGGATATCGGGGAAGAACAGTATGTCGAGCTATTTTTTGACTTACTGGGGATCAAGCCTGATTGGGAG
AAAATAAAATATTATATTTTACTGGATGAATTGTTTTAGTACCTAGATTTAGATATCTAAA***GCATGC***
*Sph*I

pACYC_P*Der (4,294 bp)

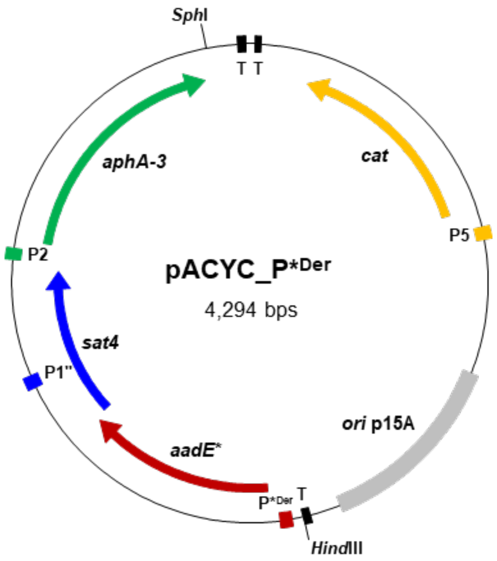

pACYC_P*Der (an insert size is 2,166 bp; a putative alternative promoter is underlined)

*Hind*III
AAGCTTGGACAAGA**TTGAAAAGTGGATAGATTTATGATACAGTGAATAGATTTATGATATAAT**GAGTTATCAACAAATCG
GAATTTGTGGAGGTAAAATATAG***ATA*****AATCAAGGTAGAATTATTGTAATCACAGGTGCGCCGGGGACAGGAAAAACTACA**
ACGGCATCTGCTGTTGCAAAAGAATCAGATTTGGAAAAGTCTGTGCATATGCACACAGATGACTTTTATCATTATCATGT
AAGAAAGCCAAGCGCAAGGGAGTATGATGATTGCTGCAATGAATTTTGGAATGTAACAGCTTATGTTATTAAAGGATTGT
GCCGTAAGGAAATTTTATTTGCTATTGATCATTTTAATCAGATTGTTCGCCATGAGCTGCTGAGAATGATATCATGGAAG

GTCGGCATCGAAACAGGCTTTAAATTAAGTGTAGGCAAGAACTATAAGTTTATTGAAAGGTATGTATCCGAGGATTTGTG
GGAGAAACTTTTGTCCACCTACCGGATGGATTCCTATGAAAACATATGGGAAGCATTATTTCTATGCCATCAATTGTTCA
GGGCGGTATCCGGTGAGGTGGCGGAATGGCTTCATTATGCCTATCCGGAGTATGATAGGAATATAACAAAATATACCAGG
GACATGTATAAAAAATACACTGGTAAAACCGGCTGCCTGGATAGCACATATGCCGCTGATATAGAAGAGAGGCGGGAACA
GTGATTACAGAAATGAAAGCAGGGCACCTGAAAGATATCGATAAACCCAGCGAACCATTTGAGGTGATAGGTAAGATTAT
ACCGAGGTATGAAAACGAGAATTGGACCTTTACAGAATTACTCTATGAAGCGCCATATTTAAAAAGCTACCAAGACGAAG
AGGATGAAGAGGATGAGGAGGCAGATTGCCTTGAATATATTGACAATACTGATAAGATAATATATCTTTACTACCAAGAC
GATAAATGCGTCGGAAAAGTTAAACTGCGAAAAAATTGGAACCGGTACGCTTATATAGAAGATATCGCCGTATGTAAGGA
TTTCAGGGGGCAAGGCATAGGCAGCGCGCTTATCAATATATCTATAGAATGGGCAAAGCATAAAAACTTGCATGGACTAA
TGCTTGAAACCCAGGACAATAACCTTATAGCTTGTAAATTCTATCATAATTGTGGTTTCAAAATCGGCTCCGTCGATACT
ATGTTATACGCCAACTTTGAAAACAACTTTGAAAAAGCTGTTTTCTGGTATTTAAGGTTTTAGAATGCAAGGAACAGTGA
ATTGGAGTTCGTC**TTGTTATAATTAGCTTCTTGGGGTATCTT**TAAATACTGTAGAAAAGAGGAAGGAAATAATAA**ATGGC**
TAAAATGAGAATATCACCGGAATTGAAAAAACTGATCGAAAAATACCGCTGCGTAAAAGATACGGAAGGAATGTCTCCTG
CTAAGGTATATAAGCTGGTGGGAGAAAATGAAAACCTATATTTAAAAATGACGGACAGCCGGTATAAAGGACCACCTAT
GATGTGGAACGGGAAAAGGACATGATGCTATGGCTGGAAGGAAAGCTGCCTGTTCCAAAGGTCCTGCACTTTGAACGGCA
TGATGGCTGGAGCAATCTGCTCATGAGTGAGGCCGATGGCGTCCTTTGCTCGGAAGAGTATGAAGATGAACAAAGCCCTG
AAAAGATTATCGAGCTGTATGCGGAGTGCATCAGGCTCTTTCACTCCATCGACATATCGGATTGTCCCTATACGAATAGC
TTAGACAGCCGCTTAGCCGAATTGGATTACTTACTGAATAACGATCTGGCCGATGTGGATTGCGAAAACTGGGAAGAAGA
CACTCCATTTAAAGATCCGCGCGAGCTGTATGATTTTTTAAAGACGGAAAAGCCCGAAGAGGAACTTGTCTTTTCCCACG
GCGACCTGGGAGACAGCAACATCTTTGTGAAAGATGGCAAAGTAAGTGGCTTTATTGATCTTGGGAGAAGCGGCAGGGCG
GACAAGTGGTATGACATTGCCTTCTGCGTCCGGTCGATCAGGGAGGATATCGGGGAAGAACAGTATGTCGAGCTATTTTT
TGACTTACTGGGGATCAAGCCTGATTGGGAGAAAATAAAATATTATATTTTACTGGATGAATTGTTTTAGTACCTAGATT
TAGATATCTAAA**GCATGC**
*Sph*I

pACYC_P*_ΔaadE* (3,791 bp)

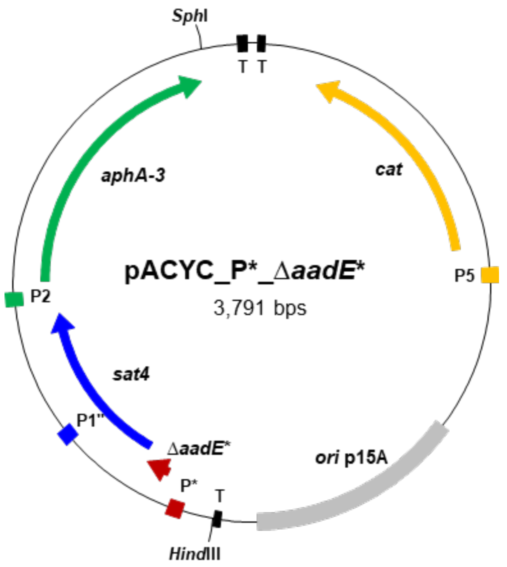

pACYC_P*_ΔaadE* (an insert size is 1,663 bp)

*Hind*III

AAGCTTGCACATTCGGTAACGGAAGCAGTTACAATCTCCTGCAAAGTCGGGAGATTTTTTCGTTATACGGTAGGTGAATG
TA**TTGAAAAAATGTAAAATTTGTGATATAAT**AAACTGAACGATAAATAAGAATTTGCGGAGGTAAAATATAG**ATGAATCA**
AGGTAGAATTATTGTAATCACAGGTGCGCCGGGGACAATAGAAGAGAGGCGGGAACAGTGATTACAGAAATGAAAGCAGG
GCACCTGAAAGATATCGATAAACCCAGCGAACCATTTGAGGTGATAGGTAAGATTATACCGAGGTATGAAAACGAGAATT
GGACCTTTACAGAATTACTCTATGAAGCGCCATATTTAAAAAGCTACCAAGACGAAGAGGATGAAGAGGATGAGGAGGCA
GATTGCCTTGAATATATTGACAATACTGATAAGATAATATATCTTTACTACCAAGACGATAAATGCGTCGGAAAAGTTAA
ACTGCGAAAAAATTGGAACCGGTACGCTTATATAGAAGATATCGCCGTATGTAAGGATTTCAGGGGGCAAGGCATAGGCA
GCGCGCTTATCAATATATCTATAGAATGGGCAAAGCATAAAAACTTGCATGGACTAATGCTTGAAACCCAGGACAATAAC

CTTATAGCTTGTAAATTCTATCATAATTGTGGTTTCAAAATCGGCTCCGTCGATACTATGTTATACGCCAACTTTGAAAA
CAACTTTGAAAAAGCTGTTTTCTGGTATTTAAGGTTTTAGAATGCAAGGAACAGTGAATTGGAGTTCGTC**TTGTTATAAT**
TAGCTTCTTGGGGTATCTTTAAATACTGTAGAAAAGAGGAAGGAAATAATAA**ATGGCTAAAATGAGAATATCACCGGAAT**
TGAAAAAACTGATCGAAAAATACCGCTGCGTAAAAGATACGGAAGGAATGTCTCCTGCTAAGGTATATAAGCTGGTGGGA
GAAAATGAAAACCTATATTTAAAAATGACGGACAGCCGGTATAAAGGGACCACCTATGATGTGGAACGGGAAAAGGACAT
GATGCTATGGCTGGAAGGAAAGCTGCCTGTTCCAAAGGTCCTGCACTTTGAACGGCATGATGGCTGGAGCAATCTGCTCA
TGAGTGAGGCCGATGGCGTCCTTTGCTCGGAAGAGTATGAAGATGAACAAAGCCCTGAAAAGATTATCGAGCTGTATGCG
GAGTGCATCAGGCTCTTTCACTCCATCGACATATCGGATTGTCCCTATACGAATAGCTTAGACAGCCGCTTAGCCGAATT
GGATTACTTACTGAATAACGATCTGGCCGATGTGGATTGCGAAAACTGGGAAGAAGACACTCCATTTAAAGATCCGCGCG
AGCTGTATGATTTTTTAAAGACGGAAAAGCCCGAAGAGGAACTTGTCTTTTCCCACGGCGACCTGGGAGACAGCAACATC
TTTGTGAAAGATGGCAAAGTAAGTGGCTTTATTGATCTTGGGAGAAGCGGCAGGGCGGACAAGTGGTATGACATTGCCTT
CTGCGTCCGGTCGATCAGGGAGGATATCGGGGAAGAACAGTATGTCGAGCTATTTTTTGACTTACTGGGGATCAAGCCTG
ATTGGGAGAAAATAAAATATTATATTTTACTGGATGAATTGTTTTAGTACCTAGATTTAGATATCTAAA***GCATGC***
*Sph*I

pACYC_P1"_ΔP2_aphA3 (3,187 bp)

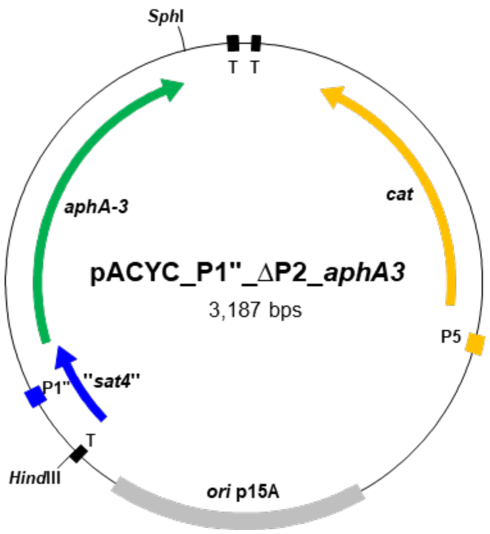

pACYC_P1"_ΔP2_aphA3 (an insert size 1,059 bp)

*Hind*III
AAGCTTACCGAGGTATGAAAACGAGAATTGGACCTTTACAGAATTACTCTATGAAGCGCCATATTTAAAAAGCTACCAAG
ACGAAGAGGATGAAGAGGATGAGGAGGCAGATTGCCTTGAATATATTGACAATACTGATAAGATAATATATCTTTACTAC
CAAGACGATAAATGCGTCGGAAAAGTTAAACTGCGAAAAAATTGGAACCGGTACGCTTATACTGTAGAAAAGAGGAAGGA
AATAATAAATGGCTAAAATGAGAATATCACCGGAATTGAAAAAACTGATCGAAAAATACCGCTGCGTAAAAGATACGGAA
GGAATGTCTCCTGCTAAGGTATATAAGCTGGTGGAGAAAATGAAAACCTATATTTAAAAATGACGGACAGCCGGTATAA
AGGGACCACCTATGATGTGGAACGGGAAAAGGACATGATGCTATGGCTGGAAGGAAAGCTGCCTGTTCCAAAGGTCCTGC
ACTTTGAACGGCATGATGGCTGGAGCAATCTGCTCATGAGTGAGGCCGATGGCGTCCTTTGCTCGGAAGAGTATGAAGAT
GAACAAAGCCCTGAAAAGATTATCGAGCTGTATGCGGAGTGCATCAGGCTCTTTCACTCCATCGACATATCGGATTGTCC
CTATACGAATAGCTTAGACAGCCGCTTAGCCGAATTGGATTACTTACTGAATAACGATCTGGCCGATGTGGATTGCGAAA
ACTGGGAAGAAGACACTCCATTTAAAGATCCGCGCGAGCTGTATGATTTTTTAAAGACGGAAAAGCCCGAAGAGGAACTT
GTCTTTTCCCACGGCGACCTGGGAGACAGCAACATCTTTGTGAAAGATGGCAAAGTAAGTGGCTTTATTGATCTTGGGAG
AAGCGGCAGGGCGGACAAGTGGTATGACATTGCCTTCTGCGTCCGGTCGATCAGGGAGGATATCGGGGAAGAACAGTATG
TCGAGCTATTTTTTGACTTACTGGGGATCAAGCCTGATTGGGAGAAAATAAAATATTATATTTTACTGGATGAATTGTTT
TAGTACCTAGATTTAGATATCTAAA***GCATGC***
*Sph*I

pACYC_P2_aphA3 (3,030 bp)

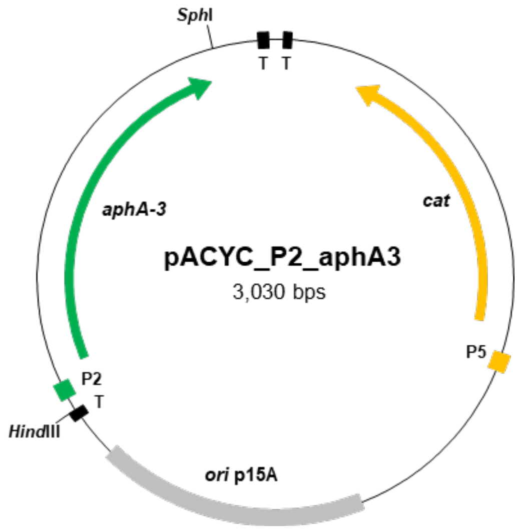

pACYC_P2_aphA3 (an insert size 902 bp)

*Hind*III
AAGCTTGGAACAGTGAATTGGAGTTCGTC**TTGTTATAATTAGCTTCTTGGGGTATCTT**TAAATACTGTAGAAAAGAGGAA
GGAAATAATAA**ATGGCTAAAATGAGAATATCACCGGAATTGAAAAAACTGATCGAAAAATACCGCTGCGTAAAAGATACG**
GAAGGAATGTCTCCTGCTAAGGTATATAAGCTGGTGGGAGAAAATGAAAACCTATATTTAAAAATGACGGACAGCCGGTA
TAAAGGGACCACCTATGATGTGGAACGGGAAAAGGACATGATGCTATGGCTGGAAGGAAAGCTGCCTGTTCCAAAGGTCC
TGCACTTTGAACGGCATGATGGCTGGAGCAATCTGCTCATGAGTGAGGCCGATGGCGTCCTTTGCTCGGAAGAGTATGAA
GATGAACAAAGCCCTGAAAAGATTATCGAGCTGTATGCGGAGTGCATCAGGCTCTTTCACTCCATCGACATATCGGATTG
TCCCTATACGAATAGCTTAGACAGCCGCTTAGCCGAATTGGATTACTTACTGAATAACGATCTGGCCGATGTGGATTGCG
AAAACTGGGAAGAAGACACTCCATTTAAAGATCCGCGCGAGCTGTATGATTTTTTAAAGACGGAAAAGCCCGAAGAGGAA

CTTGTCTTTTCCCACGGCGACCTGGGAGACAGCAACATCTTTGTGAAAGATGGCAAAGTAAGTGGCTTTATTGATCTTGG
GAGAAGCGGCAGGGCGGACAAGTGGTATGACATTGCCTTCTGCGTCCGGTCGATCAGGGAGGATATCGGGGAAGAACAGT
ATGTCGAGCTATTTTTTGACTTACTGGGGATCAAGCCTGATTGGGAGAAAATAAAATATTATATTTTACTGGATGAATTG
TTTTAGTACCTAGATTTAGATATCTAAA***GCATGC***
*Sph*I

pACYC_ΔP2_aphA3-19 (2,971 bp)

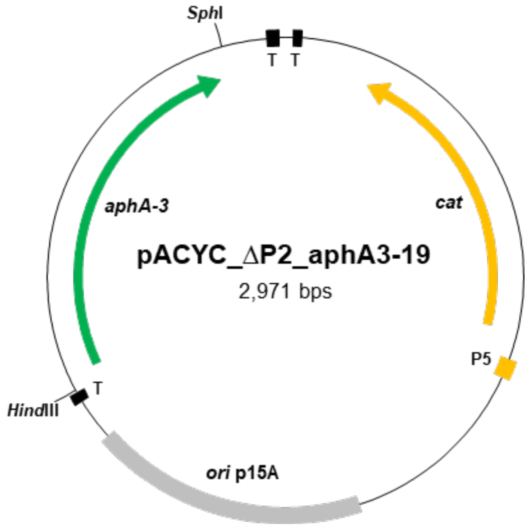

pACYC_ΔP2_aphA3-19 (an insert size 843 bp)

*Hind*III
AAGCTTTGTAGAAAAGAGGAAGGAAATAATAA**ATGGCTAAAATGAGAATATCACCGGAATTGAAAAAACTGATCGAAAAA**
TACCGCTGCGTAAAAGATACGGAAGGAATGTCTCCTGCTAAGGTATATAAGCTGGTGGGAGAAAATGAAAACCTATATTT
AAAAATGACGGACAGCCGGTATAAAGGGACCACCTATGATGTGGAACGGGAAAAGGACATGATGCTATGGCTGGAAGGAA
AGCTGCCTGTTCCAAAGGTCCTGCACTTTGAACGGCATGATGGCTGGAGCAATCTGCTCATGAGTGAGGCCGATGGCGTC

CTTTGCTCGGAAGAGTATGAAGATGAACAAAGCCCTGAAAAGATTATCGAGCTGTATGCGGAGTGCATCAGGCTCTTTCA
CTCCATCGACATATCGGATTGTCCCTATACGAATAGCTTAGACAGCCGCTTAGCCGAATTGGATTACTTACTGAATAACG
ATCTGGCCGATGTGGATTGCGAAAACTGGGAAGAAGACACTCCATTTAAAGATCCGCGCGAGCTGTATGATTTTTTAAAG
ACGGAAAAGCCCGAAGAGGAACTTGTCTTTTCCCACGGCGACCTGGGAGACAGCAACATCTTTGTGAAAGATGGCAAAGT
AAGTGGCTTTATTGATCTTGGGAGAAGCGGCAGGGCGGACAAGTGGTATGACATTGCCTTCTGCGTCCGGTCGATCAGGG
AGGATATCGGGGAAGAACAGTATGTCGAGCTATTTTTTGACTTACTGGGGATCAAGCCTGATTGGGAGAAAATAAAATAT
TATATTTTACTGGATGAATTGTTTTAGTACCTAGATTTAGATATCTAAA***GCATGC***

*Sph*I

pACYC_ΔP2_aphA3-17 (2,971 bp)

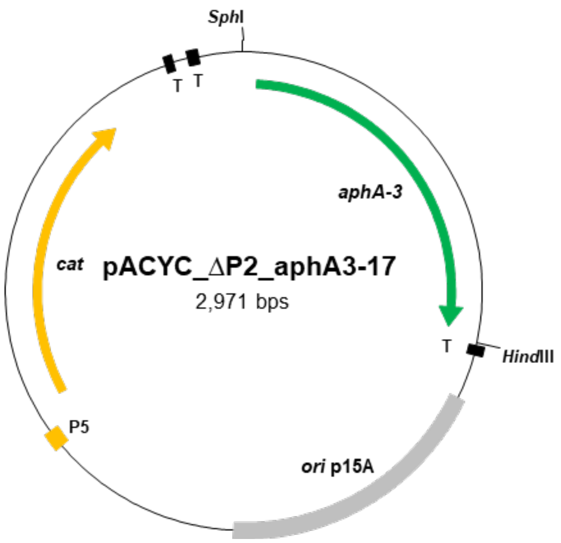

pACYC_ΔP2_aphA3-17 (an insert size 843 bp)

*Sph*I
GCATGCTGTAGAAAAGAGGAAGGAAATAATAA**ATGGCTAAAATGAGAATATCACCGGAATTGAAAAAACTGATCGAAAAA**

TACCGCTGCGTAAAAGATACGGAAGGAATGTCTCCTGCTAAGGTATATAAGCTGGTGGGAGAAAATGAAAACCTATATTT
AAAAATGACGGACAGCCGGTATAAAGGGACCACCTATGATGTGGAACGGGAAAAGGACATGATGCTATGGCTGGAAGGAA
AGCTGCCTGTTCCAAAGGTCCTGCACTTTGAACGGCATGATGGCTGGAGCAATCTGCTCATGAGTGAGGCCGATGGCGTC
CTTTGCTCGGAAGAGTATGAAGATGAACAAAGCCCTGAAAAGATTATCGAGCTGTATGCGGAGTGCATCAGGCTCTTTCA
CTCCATCGACATATCGGATTGTCCCTATACGAATAGCTTAGACAGCCGCTTAGCCGAATTGGATTACTTACTGAATAACG
ATCTGGCCGATGTGGATTGCGAAAACTGGGAAGAAGACACTCCATTTAAAGATCCGCGCGAGCTGTATGATTTTTTAAAG
ACGGAAAAGCCCGAAGAGGAACTTGTCTTTTCCCACGGCGACCTGGGAGACAGCAACATCTTTGTGAAAGATGGCAAAGT
AAGTGGCTTTATTGATCTTGGGAGAAGCGGCAGGGCGGACAAGTGGTATGACATTGCCTTCTGCGTCCGGTCGATCAGGG
AGGATATCGGGGAAGAACAGTATGTCGAGCTATTTTTTGACTTACTGGGATCAAGCCTGATTGGGAGAAAATAAAATAT
TATATTTTACTGGATGAATTGTTTTAGTACCTAGATTTAGATATCTAAA***AAGCTT***
*Hind*III

References:

1. Caillaud, F.; Trieu-Cuot, P.; Carlier, C.;Courvalin, P. Nucleotide sequence of the kanamycin resistance determinant of the pneumococcal transposon Tn*1545*: evolutionary relationships and transcriptional analysis of *aphA-3* genes. *Mol Gen Genet.* **1987,** *207*, 509-513.
2. Trieu-Cuot, P.; Gerbaud, G.; Lambert, T.; Courvalin, P. *In vivo* transfer of genetic information between gram-positive and gram-negative bacteria. *EMBO J* **1985,** *4*, 3583-3587.
3. Trieu-Cuot, P.; Klier, A.; Courvalin, P. DNA sequences specifying the transcription of the streptococcal kanamycin resistance gene in *Escherichia coli* and *Bacillus subtilis*. *Mol Gen Genet.* **1985**, *198*, 348-352.

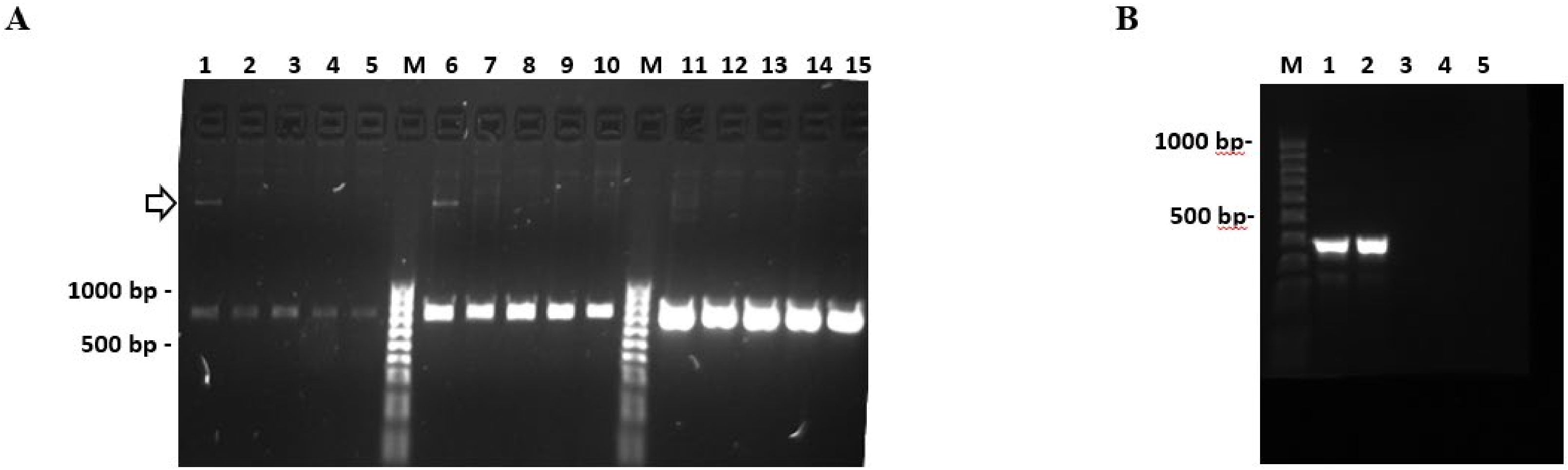

Figure S6: analyses of a potential loss of the *aadE*-sat4-aphA-3* gene cluster as well as prophage during multiple passages of *M. bovirhinis* HAZ141_2 under nonselective conditions. **(A).** Semi-quantitative PCR was performed to detect a potential increase in amount of the PCR product (727 bp in length) available upon excision of the *aadE*-sat4-aphA-3* gene cluster during nonselective serial passages (in Kn-free broth). Genomic DNA (50 ng) of *M. bovirhinis* HAZ141_2 parent strain (p.3) as well as DNAs of clonal passages were subjected to PCR amplification with 0922_down_F1 and up-aadE_F2 primers (Table S1) using different number of amplification cycles. *M. bovirhinis* HAZ141_2 p.3 (lanes 1, 6, and 11), p.30 (lanes 2, 7 and 12) and p.50 (lanes 3, 8 and 13), p. 80 (lanes 4, 9 and 14) and p.100 (lanes 5, 10 and 15). The numbers of PCR cycles were as the following: 22 – lanes 1-5; 26 – lanes 6-10; and 30 – lanes 11-15. A weaker band near 6.6 kb appeared in lanes 1 and 6 represents an intact genomic *aadE*-sat4-aphA-3* containing region present in the wild type configuration. **(B).** Potential excision of *M. bovirhinis* HAZ141_2 prophage was tested using pseudo_R and tRNA-Leu_F primers (Table S1). The PCR allows identification of the predicted restored junction upon excision of the prophage as well as a "prophage-empty place" in prophagesless *M. bovirhinis* strains. *M. bovirhinis* type strain PG43 (lane 1), Israeli *M. bovirhinis* strain 316981 (lane 2) and *M. bovirhinis* HAZ141_2 p.3, p.50 and p.100 clones (lanes 3-5, respectively). PCR product of 378 bp was obtained only in prophagesless strains (lanes 1-2). The 100-bp ladder (BioRad, California, USA) is shown as M.
