# Supplementary material for: The aadE*-sat4-aphA-3 Gene Cluster of Mycoplasma bovirhinis HAZ141_2 Undergoes Genomic Rearrangements Influencing the Primary Promoter Sequence
Source: Antibiotics (Basel). 2021 Nov 1;10(11):1335. doi: 10.3390/antibiotics10111335 (PMC8614714; doi:10.3390/antibiotics10111335)
Supplement: Supplementary file 1 [file antibiotics-10-01335-s001.zip › antibiotics-1428173-supplementary/Table_S1-S2.pdf]

Table S1: oligonucleotides used in this study.

Primer designation	Sequence (5' to 3')	Purpose of use	PCR conditions [a]
pseudo_R	TCATTAATCACCCCTTTTCC	Confirmation of a new junction, obtained after inversion *via* 197-bp IRs (Fig. 1B). The PCR product is 824 bp.	T=58º C; t=45 sec
pcp_R2	GTCCGCTCCATACGATACCG		
IS2010-0923-R	ACAGTCTTTGATATCCTAATG	Confirmation of a new junction, obtained after inversion *via* 197-bp IRs (Fig. 1B). The PCR product is 1,979 bp.	T=56º C; t=2 min
antisig_R1	CTCCAAATCATAGAGCCACC		
aadE_R3[b]	ATCATCATACTCCCTTGCGC	Confirmation of a new junction, obtained after inversion *via* 155-bp IRs (Fig. 1C); used with IS2010-0923-R. The PCR product is 1,066 bp.	T=54º C; t=45 sec
CPT_R	GCCACGGCTCTAAAAACCAC	Confirmation of a new junction, obtained after inversion *via* 155-bp IRs (Fig. 1C). The PCR product is 638 bp.	T=60º C; t=45 sec
pinE1_F1	AACAGCCACGGTTAACAGAG		
0922_down_F1	GCGTACAGTTAAACTCACAG	Restored sequence junction after deletion (excision) of the *aadE*-sat4-aphA-3* gene cluster *via* 155-bp DRs (WT; Fig. 1A) as well as after excision *via* 197-bp DRs (Fig. 1C). The PCR product is 727 bp. In addition, these primers were used in semi-quantitative PCR to test excision of the *aadE*-sat4-aphA-3* cluster during nonselective serial passages (Fig. S6A).	T=56º C; t=30 sec
up-aadE_F2	GCACATTCGGTAACGGAAGC		
0926_197_DR_R1	ACAAGAGTGGTGCGCTTAC	Used with aadE_R3 to detect a circularization of the *aadE*-sat4-aphA-3* genes-containing region occurred *via* 155-bp DRs (WT; Fig. 1A) as well as *via* 197-bp DRs (Fig. 1C). The PCR product is 2,921 bp.	T=59º C; t=1 min 45 sec
tRNA_Leu_F	AGTGGTGAAATTGGCAGAC	Used with pseudo_R to detect excision of the prophage during multiple	T=59º C; t=15 sec

		passages under non-selective conditions (Fig. S6B). In the prophageless strains obtained PCR product is of 378 bp.	
IS1_del_For	**GCGGAGTGTATACTGGCTTAC**	Amplification of the pACYC184 derivate plasmid without *tet* gene as well as P2 and P4 promoters (pACYC_Δtet $\Delta P2^{vec}\Delta P4^{vec}$). First step PCRs: IS1_del_For was used with NEW_HindIII_pACYC184, while IS1_del_Rev was used with pACYC184-R1-SphI. Second step assembly PCR was performed with NEW_HindIII_pACYC184 and pACYC184-R1-SphI using PCR products obtained in the first step.	First PCRs (both reactions): T=58° C; t=1min 30 sec. Second PCR: T=60° C; t=2min
NEW_HindIII_pACYC184	attaAAGCTTACATGAGAATTACAAC		
IS1_del_Rev	**GTAAGCCAGTATACACTCCGC** CGACGCACTTTGCGCCGAA		
pACYC184-R1-SphI[b]	tataGCATGCGTATTAACGAAGCGCTAACC		
woP2_aphA_SphI	attaGCATGCTGTAGAAAAGAGGAAG	Amplification of the P2-promoterless *aphA-3* gene to clone into pACYC_Δtet $\Delta P2^{vec}\Delta P4^{vec}$ in the same direction as *cat* gene (pACYC_ΔP2_aphA3-17). The PCR product is 855 bp.	T=56° C; t=45 sec
aphA3-R1-HindIII[b]	attaAAGCTTTTTAGATATCTAAATCTAGG		
woP2_aphA_HindIII	attaAAGCTTTGTAGAAAAGAGGAAG	Amplification of the P2-promoterless *aphA-3* gene to clone into pACYC_Δtet $\Delta P2^{vec}\Delta P4^{vec}$ in direction opposite of *cat* gene (pACYC_ΔP2_aphA3-19). The PCR product is 855 bp.	T=56° C; t=45 sec
down_aphA-R3_SphI	attaGCATGCTTTAGATATCTAAATCTAGG		
up_aadE-F3_HindIII	attaAAGCTTGCACATTCGGTAACGGAAGC	Amplification of the *aadE*-sat4-aphA-3* gene cluster located under P^{*} promoter to clone into pACYC_Δtet $\Delta P2^{vec}\Delta P4^{vec}$ in direction opposite of *cat* gene (pACYC_P*); used with down_aphA-R3_SphI. The PCR product is 2,227 bp.	T=56° C; t=2 min

aadE-F3-HindIII	tataAAGCTTGGACAAGATTGAAAAGTGG	Amplification of the *aadE*-sat4-aphA-3* gene cluster under P^{*Der} promoter, obtained after inversion through 155-bp IRs (Fig. 1C) to clone into pACYC_Δtet $\Delta P2^{vec}\Delta P4^{vec}$ in direction opposite of *cat* gene (pACYC_P^{*Der}); used with down_aphA-R3_SphI. The PCR product is 2,178 bp;	T=56° C; t=2 min
P2_For1_HindIII	tataAAGCTTGGAACAGTGAATTGGAG	Amplification of the *aphA-3* gene under P2 promoter to clone into pACYC_Δtet $\Delta P2^{vec}\Delta P4^{vec}$ in direction opposite of *cat* gene (pACYC_P2_aphA3); used with down_aphA-R3_SphI. The PCR product is 914 bp.	T=56° C; t=1 min
aadE_del_5'Rev	**CTGTTCCCGCCTCTCTTCTAT**TGTCCCC GGCGCACCTGTG	Amplification of the *sat4-aphA-3* genes to clone into pACYC_Δtet $\Delta P2^{vec}\Delta P4^{vec}$ in direction opposite of *cat* gene; in this construct the *aadE** gene was deleted (pACYC_P*_Δ aadE*). First step PCRs: aadE_del_5'Rev was used with up_aadE-F3_HindIII, while aadE_del_3'For was used with down_aphA-R3_SphI. Second step of assembly PCR was performed with up_aadE-F3_HindIII and down_aphA-R3_Sph using PCR products obtained in the first step. The PCR product is 1,675 bp.	First PCRs: T=60° C; t=15 sec or t=1 min 30 sec, respectively. Second PCR: T=60° C; t=1 min 30 sec.
aadE_del_3'For	**ATAGAAGAGAGGCGGGAACAG**TG		
sat4_F3_Hind	tataAAGCTTACCGAGGTATGAAAACGAG	Amplification of the *aphA-3* gene under P1" with deletion of most of the *sat4* gene as well as P2 promoter of the *aphA-3* gene (pACYC_P1"_ΔP2_aphA3). First step PCRs: sat4_F3_Hind was used with	First PCRs: T=58° C; t=15 sec or t=45 sec, respectively. Second PCR: T=60° C; t=1 min.
sat4-R2[b]	**TATAAGCGTACCGGTTCC**		
aph_Pdel_For	AATT**GGAACCGGTACGCTTATA**CTGTA GAAAAGAGGAAGG		

	sat4-R2, while aph_Pdel_For was used with down_aphA-R3_SphI. Second step of assembly PCR was performed with sat4_F3_Hind and down_aphA-R3_SphI using PCR products obtained in the first step. The PCR product is 1,071 bp.

[a] T – annealing temperature; t – amplification time; number of amplification cycles were either 30 or 35.
[b] Sequence of these primers have been previously published [1].
*Hind*III and *Sph*I sites are underlined, while 5' nucleotides, added to improve restriction cleavage, are shown as lowercase letters.
Complementary sequences present in the primers used in the assembly PCRs are bolded.
When needed, internal primers were used to confirm the sequences obtained (data not shown).
The size of the *aadE*-sat4-aphA-3* gene-related PCR constructs includes *Hind*III and *Sph*I sites.

References

1. Lysnyansky, I.; Borovok, I. A GC-rich prophage-like genomic region of *Mycoplasma bovirhinis* HAZ141_2 carries a gene cluster encoding resistance kanamycin and neomycin. *Antimicrob Agents Chemother* **2021**, *65*:e01010-20. doi: 10.1128/AAC.01010-20.

Table S2: description of the recombinant plasmids used in this study.

Plasmid construct[1]	Description of the construct
pACYC_ΔtetΔP2^{vec}ΔP4^{vec}	Derivate of pACYC184 plasmid without *tet* gene as well as P2 and P4 promoters (Fig. S4).
pACYC_ΔP2_aphA3-17	P2-promoterless *aphA-3* gene (Fig. S5) cloned into pACYC_ΔtetΔP2^{vec}ΔP4^{vec} plasmid in direction of the *cat* gene.
pACYC_ΔP2_aphA3-19	P2-promoterless *aphA-3* gene (Fig. S5).
pACYC_P*	The *aadE**-*sat4*-*aphA-3* gene cluster is under P* promoter, located upstream of *aadE** gene (Fig. 1A and Fig. S5).
pACYC_P*^{Der}	The *aadE**-*sat4*-*aphA-3* gene cluster is under derivate (Der) P* promoter, obtained after inversion through 155-bp IRs (Fig. 1C, Fig. 2, and Fig. S5).
pACYC_P*_Δ aadE*	The *sat4*-*aphA-3* genes are under P* promoter; the *aadE**gene is deleted (Fig. S5).
pACYC_P1"_ΔP2_aphA3	The *aphA-3* gene is under P1" promoter, located within the *sat4* gene. In this construct the P*, *aadE** and P2 sequences were deleted. In addition, in this construct 5'- as well as 3'-encoding sequences of *sat4* gene were deleted remaining mostly the P1"-*sat4* containing region (Fig. S5).
pACYC_P2_aphA3	The *aphA-3* gene is under P2 promoter (Fig. S5).

[1] All constructs were cloned into pACYC_ΔtetΔP2^{vec}ΔP4^{vec} derivate in orientation opposite of the *cat* gene, otherwise indicated.
